# Cardiolipin Modulates the Activity of the Intramembrane Protease GlpG Inducing Membrane Restructuring

**DOI:** 10.1021/jacs.6c09491

**Published:** 2026-08-27

**Authors:** Barbara Maier, Oskar Engberg, Viola Döbel, Jenny Leopold, Holger A. Scheidt, Rosalie A. M. van Beekveld, Markus Weingarth, Albert A. Smith, Daniel Huster

**Affiliations:** † 9180 Institute of Medical Physics and Biophysics, Härtelstr. 16/18, Leipzig 04107, Germany; ‡ 8125 Universiteit Utrecht, NMR, Padualaan 8, Utrecht 3584 CH, The Netherlands

## Abstract

Cardiolipin (CL) is a very important lipid in bacteria and the mitochondria of higher cells. CL is characterized by a unique structure, featuring four acyl chains and two negative charges, exhibiting a highly negative intrinsic curvature. We investigate the influence of CL on the function of the intramembrane *E. coli* protease GlpG, reconstituted into *E. coli*-like membranes. To underline the importance of CL, we find a ∼40% accelerated substrate cleavage rate in the presence of 10 mol % CL in phosphatidylethanolamine (PE)/phosphatidylglycerol (PG) membranes. CL induces a lateral restructuring of the membrane: without CL, PE/PG in the lipid annulus show a decreased chain length to match the hydrophobic thickness of GlpG. When CL is present, PE and PG share the properties of the bulk lipids, while CL undergoes the same amount of thinning that was observed for PE/PG before. To reveal the molecular properties of CL, we collect a multitude of NMR parameters to quantitatively describe the structure and dynamics of CL in the absence and presence of GlpG. In addition to the chain disordering and reduction of membrane thickness, we observe that some CL species follow the slow rotational diffusive motions of GlpG. This implies an association with GlpG, indicating preferential GlpG-CL interactions. We suggest that the high negative charge and the negative intrinsic curvature of CL are the main determinants of this remarkable behavior.

## Graphical abstract

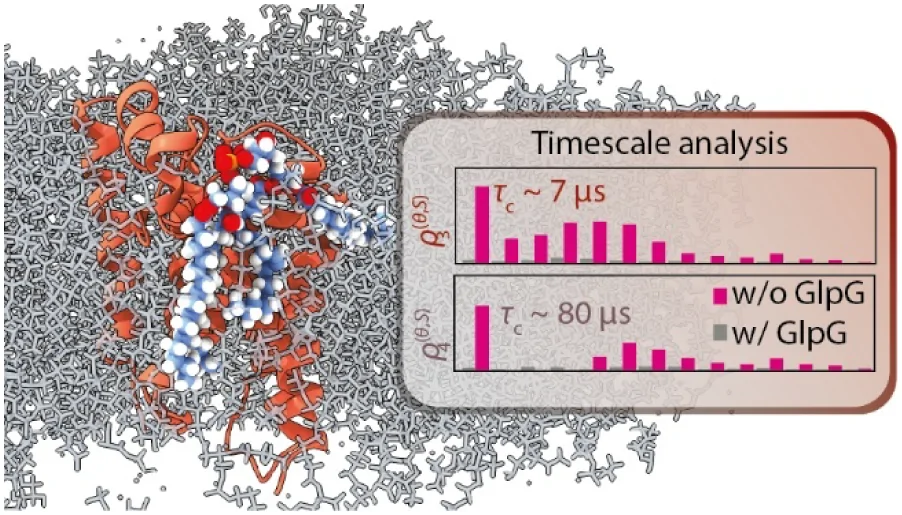

## Introduction

Chemical diversity is key to all biological reactions and life in general. This heterogeneity that amounts to up to one million different protein isoforms in the mammalian proteome represents the machinery that drives all biological processes.^1^ On the order of 10,000 small endogenous molecules modulate proteins by direct or allosteric mechanisms. For instance, more than 800 G protein-coupled receptors are activated by thousands of chemically highly diverse molecules triggering specific signal transduction cascades. This high specificity is also key to the action of drugs which exploit the chemical variability to finetune the affinity of these molecules toward their target structures.^2^ But chemical diversity comes at a price: a cell has to maintain a multitude of biochemical synthetic pathways against the evolutionary pressure that aims to find the most efficient and simple principle to execute function. While the high chemical diversity of ligands, hormones or other biological modulators is required for unique binding poses at the target proteins, the high complexity and chemical diversity of the lipidome in cellular membranes, which are the most important interfaces in biology, remains enigmatic. More than 1000 different lipids constitute the plasma membrane, the biological reason for this diversity is largely unknown.^3^ Furthermore, membranes preserve a high degree of lateral and transversal heterogeneity and it requires enormous energetic resources to maintain this entropically unfavorable state.^4–6^ Why does a cell maintain such a high chemical diversity of the lipidome that seems to contradict evolutionary and thermodynamic principles? The simple answer is we do not know.

A role of specific lipids in the modulation of protein function has only been described for a few species. The requirement of a specific environment for optimal function of some membrane proteins has been established. For instance, the rod outer segment disk membranes hosting rhodopsin are rich in docosahexaenoic acid (DHA).^7^ While rhodopsin also functions when reconstituted into saturated phosphatidylcholine (PC) membranes, its activation equilibrium is only comparable to native disk membranes when reconstituted into DHA-rich bilayers.^7^ Also, phosphatidylethanolamine (PE) featuring a negative intrinsic curvature is an important modulator of rhodopsin.^8^ Polyphosphorylated phosphoinositides like PIP_2_ are also known to modulate protein function.^9–11^ Sphingolipids, such as ceramides and gangliosides regulate protein signaling and neuronal plasticity,^12^ along with the ubiquitous cholesterol.^13^

When discussing the role of lipids in protein function, two aspects have to be considered. The modulatory role of lipids could arise from (*i*) specific lipid–protein interactions or (*ii*) the general membrane properties such as hydrophobic thickness, curvature, dynamics, electrostatics, tension or lateral pressure. Examples for either mechanism are described in the literature. A recent study compared the role of different species of negatively charged phospholipids on the activation of the A_2A_ adenosine G protein-coupled receptor (A_2A_AR).^14^ In POPC nanodiscs, only ground and intermediate states of A_2A_AR were populated. When only 5 mol % of the highly negatively charged PIP_2_ was added, the conformational ensemble was shifted toward the activated state suggesting a highly specific interaction of A_2A_AR with PIP_2_. However, the same shift in the population of the active state was observed when 30 mol % of singly negatively charged lipid was added irrespective of lipid headgroup chemistry. This shows that while highly specific interactions between PIP_2_ exist a similar protein activation can also be induced by more bulk-like membrane properties such as membrane surface potential. The impact of cholesterol on protein function can also be considered from these two perspectives. On the one hand, tightly bound cholesterol has been found in numerous crystal and cryo-EM structures of membrane proteins. On the other hand, cholesterol exerts a very pronounced condensation effect onto lipid chains leading to a strong increase in membrane thickness, suggesting that the bulk membrane properties are key to the modulation of proteins.^15^

In recent work, we studied the activation of the small rhomboid protease GlpG from *E. coli* in lipid membranes of varying composition.^16,17^ GlpG is a small serine protease that cleaves intramembrane substrates. We found that the lipid environment had a strong effect on the activity of the protease. Remarkably, proteolysis was fastest in artificial DMPC or POPC membranes, representing lipids that are not found in *E. coli*. In these membranes, GlpG had no influence on lipid chain order or headgroup orientation and dynamics. However, in membranes of an *E. coli*-like lipid mix of POPE/POPG, the presence of GlpG induced a strong decrease in lipid chain order inducing a thinning of the surrounding lipids to match the hydrophobic thickness of the protein. Also, a strong alteration in headgroup orientation and dynamics was observed.^17^

In our previous work, an important lipid component of *E. coli* was missing, cardiolipin (CL). CL is found in small proportions (5–10%) in bacterial and mitochondrial membranes.^18^ Its structure features four acyl chains, which is the reason why it is also termed “double” phospholipid,^19^ and two negatively charged phosphate groups connected by a glycerol moiety (Figure 1A). CL has a high negative intrinsic curvature, promoting an inverse hexagonal (H_II_) phase.^19^ CL’s regulatory activity for bacterial and mitochondrial proteins has been highlighted.^20,21^ Here, we investigate GlpG function in an *E. coli*-lipid mix containing CL and the interaction of GlpG with that specific lipid. CL not only modulates the activity of GlpG, it is also found that CL preferentially interacts with the protein suggesting an allosteric modulation of protein function.

**Figure 1:**
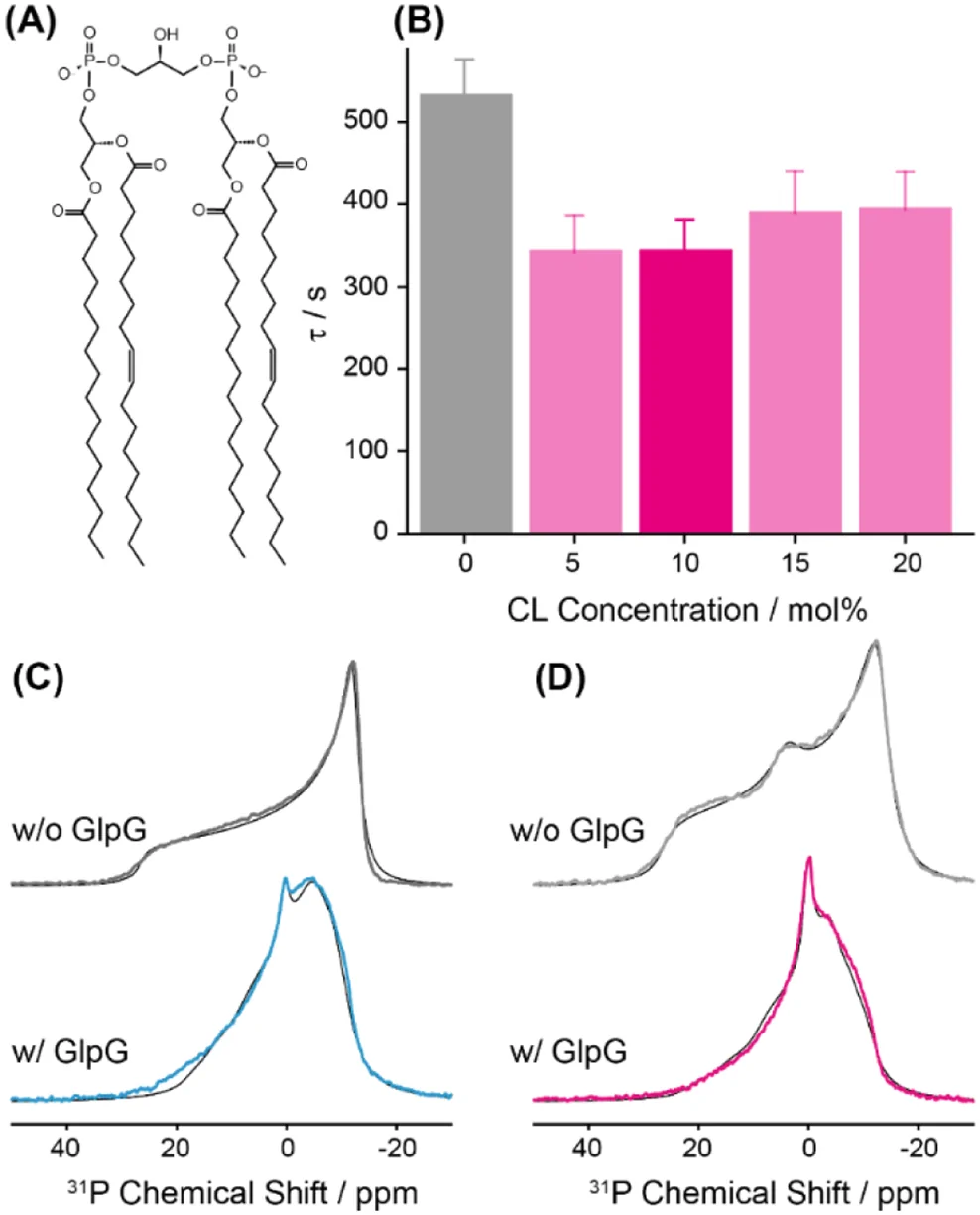
Effect of CL on the activity of GlpG and lipid headgroup perturbation by CL and GlpG. (A) chemical structure of a typical CL with two palmitoyl and two oleoyl chains. (B) The characteristic GlpG cleavage time constant for proteolysis (τ) of the substrate LacYTM2 is plotted as a function of the CL content of POPE/POPG (75:25) membranes. Errors represent the standard deviation of three biological replicates. (C)^31^P NMR spectra of POPE/POPG (molar ratio 75/25) in the absence (top) and in the presence of GlpG (bottom). (D) ^31^P NMR spectra of POPE/POPG/CL (molar ratio 67.5/22.5/10) in the absence (top) and in the presence of GlpG (bottom) at an original protein to lipid molar ratio of 1:125. NMR spectra were measured at a temperature of 37 °C. Thin black lines represent best fit line shape calculations. We analyzed the protein:lipid ratio in the samples using TLC and protein concentration measurements (supplementary Figure S2). Samples contained between 47 and 78 lipids per protein, which is somewhat below the original mixing ratio of 1:125. A similar behavior has also been observed in previous experiment.^17^.

## Materials and Methods

### Materials

The peptide substrate LacYTM2­(EDANS/DABCYL) (sequence KRHDINE­(EDANS)ISK SDTGK­(DABCYL)IFAAI SLFSLLFQPL FGLLSKK) was synthesized by the Core Unit Peptide Technologies at the University of Leipzig using standard Fmoc solid phase peptide synthesis. The lipids 1,2-diheptanoyl-*sn*-glycero-3-phosphocholine (DHPC), 1-palmitoyl-2-oleoyl-*sn*-glycero-3-phosphoethanolamine (POPE), POPE-*d*_31_ (perdeuterated in the palmitoyl chain), 1-palmitoyl-2-oleoyl-*sn*-glycero-3-phospho-(1′-rac-glycerol) (POPG), and 1′,3′-bis­[1-palmitoyl-2-oleoyl-*sn*-glycero-3-phospho]-glycerol (sodium salt) (CL) were purchased from Avanti Polar Lipids (Alabaster, AL, USA). n-Dodecyl-β-D-maltoside (DDM) was purchased from Glycon (Luckenwalde, Germany). Unless otherwise specified, all additional reagents were analytical grade or higher. Solvents used for mass spectrometry were MS grade, while all other experiments were performed using HPLC-grade solvents.

### Preparation and Analytics of U–^13^C-labeled CL

^13^C-labeled CL was isolated as described previously.^22^ *E. coli* JW3596 was grown overnight in 100 mL LB in a 250 mL baffled flask at 37 °C, 220 rpm shaking. Cells were diluted to OD_600_ = 0.05 in 4 × 1 L M9 medium containing 2 g/L U–^13^C–D-Glc and 1 g/L ^15^NH_4_Cl in 2 L baffled flasks. Bacteria were incubated for 16 h under the same conditions, harvested, pooled and freeze-dried. The dry cells were resuspended in 600 mL PBS, 5 mM EDTA, followed by the addition of 1500 mL MeOH and 750 mL CHCl_3_. The mixture was vigorously steered for 60 min, before 750 mL CHCl_3_ and 750 mL PBS, 5 mM EDTA were subsequently added to convert the system into two phases. The lower organic phase was collected, dried and redissolved in 100 mL CHCl_3_. The lipidic mixture was loaded on a column (silica 60, 0.040–0.063 mm) and phospholipids were isocratically eluted with CHCl_3_/EtOH/NH_3_/H_2_O 48/48/1/3 supplemented with 0.2 g/L NH_4_OAc.

For mass spectrometry analysis (supplementary Figure S1), the evaporated (unlabeled) CL fraction was dissolved in methanol (MA grade, Biosolve BV, Valkenswaard, The Netherlands) to a concentration of 100 μg/mL and analyzed by electrospray ionization mass spectrometry (ESI MS) after direct infusion. The source conditions at the AmaZon SL (Bruker, Bremen, Germany) were as follows: dry temperature −200 °C; dry gas −3.0 L/min; nebulizer gas −7.3 psi; capillary voltage −4500 V; end plate offset −500 V; ionization mode—negatively ionization; trap conditions – enhanced resolution. CL species were collected in the ion trap and scanned either after 35,000 target ions or after 50 ms of accumulation time using the ion count control (ICC) mode. The negatively charged ions were then identified according to their mass-to-charge (*m*/*z*) ratio using the software DataAnalysis (Bruker) if the signal-to-noise ratio (*S/N*) was higher than 3. The formation of doubly charged ions was favored compared to singly charged ions.

### Recombinant Expression of GlpG-His6

A 100 μL aliquot of chemically competent C43 (DE3) cells was thawed on ice and transformed with the pET-25b (+) *E. coli* expression vector (Novagen), carrying the sequence of wild type *E. coli* GlpG. Briefly, 100 ng of plasmid DNA was added and the cells were incubated on ice for 30 min, followed by heat shock of 2 min at 42 °C and recovery on ice for another 2 min. Subsequently, 900 μL of super optimal broth medium was added and the cells were incubated for 1 h at 37 °C with shaking before plating on LB agar plates, supplemented with 100 μg/mL ampicillin, and overnight incubation at 37 °C. After 12–16 h, a single colony was used to inoculate a 100 mL preculture of lysogeny broth (LB) medium containing 100 μg/mL ampicillin and grown overnight at 37 °C with shaking. After 16–20 h, 20–25 mL of the preculture was used to inoculate 1 L of LB medium with 100 μg/mL ampicillin to obtain a starting OD_600_ of 0.05–0.08 and then incubated at 37 °C while shaking. In total, a 3 L culture volume was prepared. Plasmid DNA from 5 mL of the remaining preculture was isolated using the NucleoSpin Plasmid kit (Macherey Nagel, Düren, Germany) and verified by Sanger sequencing (Microsynth Seqlab, Göttingen, Germany). The OD_600_ of the main culture was monitored hourly. At OD_600_ 0.8–1, protein expression was induced by addition of 0.4 mM IPTG and the culture was incubated for 16–20 h at room temperature (20– 24 °C). Cells were harvested by centrifugation at 6000 × g for 15 min at 4 °C and resuspended in ∼ 50 mL PBS per 1 L culture pellet. Two mM MgCl_2_, 0.2 mg/mL lysozyme, and 10 μg/mL DNase I were added and the cells were incubated on ice for 30–60 min. Cell disruption was performed by high-pressure homogenization at 1000 bar for 5–7 cycles using an APV 2000 homogenizer (Axflow, Düsseldorf, Germany). Cell debris were removed by centrifugation at 10,000 × g at 4 °C for 30 min. Membranes were collected by ultracentrifugation of the supernatant at 100,000 × g for 2 h at 4 °C. The membrane pellet was solubilized in 90–100 mL solubilization buffer (25 mM HEPES pH 7.4, 150 mM NaCl, 10% glycerol (v/v), 1.5% DDM (w/v)) using a Potter–Elvehjem homogenizer (VWR Avantor, Gliwice, Poland) and an overhead stirrer EUROSTAR 20 (VWR Avantor, Gliwice, Poland) until the membranes were completely dispersed. The membrane fraction was incubated overnight at 4 °C on a stirring wheel. Insoluble material was removed by ultracentrifugation at 100,000 × g for 1 h at 4 °C.

Protein purification was done by immobilized metal affinity chromatography using TALON metal affinity resin (Takara Bio, Saint-Germain-en-Laye, France). After equilibration of the resin with water and solubilization buffer, the supernatant was incubated with resin (3 mL slurry per 1 L culture) for at least 2 h at 4 °C with agitation. The resin-protein was loaded onto a column and the flow through was collected. Three washing steps were carried out with each 10 column volumes (CV) of buffer (25 mM HEPES pH 7.4, 150 mM NaCl, 10% glycerol (v/v), 0.1% DDM (w/v) and 0, 5, 10 mM imidazole in ascending order). Protein was eluted with 4 CV elution buffer (25 mM HEPES, pH 7.4, 150 mM NaCl, 10% glycerol (v/v), 0.1% DDM (w/v), 150 mM imidazole). Samples from flow through, washing steps and elution were collected for SDS-PAGE analysis. Imidazole was removed by dialysis using a 12–14 kDa MWCO dialysis tube (SpectraPor, Carl Roth, Karlsruhe, Germany) against at least 3 L of dialysis buffer (25 mM HEPES pH 7.4, 150 mM NaCl, 0.1% DDM (w/v)) for 3 h at 4 °C.

Protein concentration was determined by NanoDrop spectrometer (NanoDrop Technologies, Wilmington, DE, USA) at 280 nm. Buffer exchange and sample concentration were performed using an Amicon stirring cell (Merck, Darmstadt, Germany) equipped with a 10 kDa MWCO cellulose membrane filter (Merck, Darmstadt, Germany). The dialysis buffer was replaced stepwise by a DDM pH 4 buffer (50 mM Na-acetate, 150 mM NaCl, 0.05% DDM, pH 4.5 (w/v)) and the samples were concentrated to approximately 0.5 mg GlpG per mL. The final concentration was determined again by NanoDrop at 280 nm.

### Membrane Preparation and GlpG Reconstitution

Lipid mixtures were prepared in chloroform/methanol solution (1:1 v/v) and dried using a rotary evaporator. Membrane preparations without GlpG were prepared by dissolving the lipid film in cyclohexane and overnight freeze-drying followed by hydration to 50 wt % buffer (50 mM NaAcetate, 150 mM NaCl, pH 4). To obtain multilamellar vesicles (MLV) suitable for solid-state NMR, the samples were freeze–thawed 10 times between LN_2_ and a 50 °C water bath. After this, the samples were inserted into 3.2 mm MAS rotors and stored at −20 °C until measurements. The GlpG-containing samples were after solvent evaporation dispersed in pH 4 buffer (50 mM NaAcetate, 150 mM NaCl) to 10 mg/mL lipid concentration and subsequently extruded through two stacked 100 nm polycarbonate membranes (Lipex Biomembranes, Vancouver, Canada) to generate large unilamellar vesicles (LUV).^23^ Bicelles were formed by adding DHPC at a 6-fold molar excess relative to lipid, followed by vortexing and incubation at 50 °C for 30 min to obtain a transparent bicelle solution. For the substrate cleavage assays, the fluorescent peptide substrate LacYTM2 (EDANS/DABCYL) was dissolved in 1 mg/mL buffer (50 mM NaAcetate, 150 mM NaCl, pH 4) containing 10 mg/mL DHPC and 0.05 wt % DDM and added after bicelle formation. GlpG, solubilized in DDM elution buffer (50 mM NaAcetate, 150 mM NaCl, 0.05% DDM, pH 4.5), was introduced into the bicelle mixture. Samples were diluted to 0.5–1 mg/mL GlpG if needed by pH 4 buffer (50 mM NaAcetate, 150 mM NaCl, 0.05 wt % DDM). An aliquot of GlpG was added to reach a lipid:GlpG molar ratio of 125:1 for NMR samples and 250:1 for the cleavage assay. Protein and substrate incorporation were facilitated by three heat-ice cycles (20 min at 42 °C followed by 20 min on ice). Detergent removal and MLV formation were achieved by adding 100 mg/mL BioBeads SM-2 from Bio-Rad and incubating at 4 °C for 4 h, followed by a second application of BioBeads and overnight incubation. Typically, two BioBead treatments were sufficient to induce MLV formation. The BioBeads were removed by pressing the solution through a 100 μm EASYstrainer filter (Greiner, Frickenhausen, Germany) into a 50 mL Falcon tube, performed on ice. The samples were centrifuged (20 min, 4,000 × g) at 4 °C. After discarding the supernatant, the pellets were resuspended with 1 mL buffer (50 mM NaAcetate, 150 mM NaCl, pH 4) and transferred to 1.5 mL Eppendorf tubes and centrifuged again (20 min at 21500 × g). The supernatant was discarded. NMR samples were packed into inert 3.2 mm MAS rotors, while kinetic substrate cleavage assay samples were stored at −20 °C until measurements.

### Static ^2^H NMR Spectroscopy and Analysis of Lipid Chain Order

Static ^2^H NMR spectra were recorded on a Bruker Avance NEO 700 MHz spectrometer (Bruker BioSpin, Rheinstetten, Germany) equipped with an H/X/F MAS probe operated under nonspinning conditions. Experiments were performed at a ^2^H Larmor frequency of 107.5 MHz. Spectra were acquired using a phase-cycled quadrupolar echo pulse sequence^24^ consisting of two π/2 pulses (∼3 μs) separated by a 30 μs delay. The recycle delay was 1 s, and the spectral width was ±250 kHz at 37 °C. Spectral processing and quantitative analysis were performed using in-house routines implemented in Mathcad (Parametric Technology Corporation, Needham, MA, USA) as described previously.^25^ ^2^H powder spectra were dePaked prior to calculation of smoothed chain order parameters (Table S1).^26^ Projected acyl chain lengths were determined using the mean-torque model.^27^ Longitudinal relaxation times (*T*_1Z_) were measured using an inversion recovery quadrupolar echo sequence with delay increments of 0.001, 0.007, 0.015, 0.03, 0.07, 0.12, 0.18, 0.25, 0.5, 1, and 2.5 s, employing a relaxation delay of 2.5 s. *R*_Z1_ relaxation rates were obtained by numerical fitting of the powder spectra using custom Mathcad scripts as previously described.^28^ Experimental line shapes were modeled using 15 Pake doublets and, where required, an additional isotropic component. Spin–lattice relaxation times were extracted from fits to the inversion recovery data for each resolved spectral component.

### Stationary ^31^P NMR Measurements

Samples were analyzed by static ^31^P NMR spectroscopy on a Bruker Avance III 600 MHz spectrometer equipped with a solution XY PASEX probe at a resonance frequency of 242.9 MHz. Spectra were recorded using the Hahn echo pulse sequence with a 90° pulse length of 10 μs and an echo delay of 50 μs. The spectral width was set to 50 kHz with a recycle delay of 3 s with low-power broadband ^1^H decoupling during acquisition (ω_H_/2π = 2.5 kHz) at 37 °C. Lineshape calculations were performed using custom routines implemented in Mathcad to determine the chemical shift anisotropy (CSA, Δσ), relative phase contributions, and possible vesicle orientation. Owing to the anisotropic magnetic susceptibility of lipid bilayers, partial alignment of membranes with the bilayer normal preferentially oriented perpendicular to the external magnetic field was occasionally observed, resulting in slight vesicle deformation.^29^ GlpG-containing samples were modeled using three axially symmetric components and one isotropic contribution, whereas control samples were adequately described by one or two axially symmetric component and one isotropic component.

### ^13^C Relaxation and DIPSHIFT Experiments and Detector Analysis

Detector analysis was performed based on ^13^C relaxation and DipShift measurements, including *T*_1_ at 400, 600, and 700 MHz ^1^H frequency, ^1^H–^13^C heteronuclear nuclear Overhauser effect (NOE) at 600 and 700 MHz, and *T*_1ρ_ at 700 MHz and 5 kHz MAS frequency, with spin-lock strengths of ∼8, 12, and 22 kHz, chosen to avoid rotational resonance recoupling (R^3^) conditions.

*T*_1_ experiments were acquired based on a saturation-recovery scheme, with ^13^C saturation followed by a recovery period, with variable delay, acquired under ^1^H saturation to suppress multiexponentiality arising from ^1^H–^13^C heteronuclear NOE transfer. *T*_1ρ_ experiments were performed by a simple π/2 pulse applied to ^13^C followed by a ^13^C spin-lock, also with variable length. *T*_1ρ_ experiments were acquired at 2–3 offsets for the spin-lock, such that the effective spin-locking field $\left(\nu_{eff}={\left(\nu_{1}^{2}+\nu_{off}^{2}\right)}^{1/2}\right)$ did not vary significantly from the desired applied field (3 offsets for 7 kHz experiments, 2 offsets for 12 and 22 kHz experiments). Heteronuclear (^1^H–^13^C) NOE experiments are recorded as steady-state enhancements, given by the ratio of ^13^C signal intensities without and with ^1^H saturation, after a long equilibration time (10–13 s, supplementary Table S2). As with *T*_1_ and *T*_1ρ_ experiments, DipShift experiments were acquired without polarization transfer, and without homonuclear decoupling during the ^1^H–^13^C dipolar evolution period. Omission of homonuclear decoupling increases modulation depth of the DipShift curves, improving their accuracy. Since ^1^H–^1^H couplings are relatively small, no significant curve distortion due to ^1^H modulation during recoupling is therefore expected. Further experimental details are found in supplementary Table S2.

Relaxation rate constants were extracted via simultaneous fitting of all spectra in each data set, with the INFOS^30^ software in MATLAB. The spectrum assignment and a reference fit are given in Figure S3. Then, each peak in the spectrum is fitted by a chemical shift, a line width, an amplitude, and a relaxation rate; the relaxation rate we extract for further analysis. Over the series of spectra, monoexponential functions characterized by the relaxation times are fitted (exp­(−t × *R*_1_ *_ρ_*) for *R*_1ρ_ relaxation, 1–exp­(−t × R_1_) for *R*_1_ relaxation). A similar approach is taken for DipShift experiments, where the series of spectra were fitted against calculated DipShift curves with varying order parameter (where *S*, the order parameter, scales the dipolar coupling, with D_obs_ = S × D_rigid_). Here, *D*_rigid_ is defined as 21.5 kHz. For ^13^C bound to 2 or 3 ^1^H, calculated curves considered multiple ^1^H, where the all ^1^H–^13^C dipolar coupling tensors were assumed to be collinear due to symmetric motion about the lipid normal.

Error for the relaxation rate constants and order parameters was extracted via bootstrapped data sets. A bootstrapped data set is created using the replicate data sets, where we randomly resample the spectra acquired. We select the same number of spectra as in the original data set, but with repetitions of some spectra and omission of others. The newly created data set is refit. This process is then repeated 240 times, where standard deviations of the fit parameters are calculated from the 240 bootstrapped data sets. Extracted rate constants and standard deviations are found in supplementary Table S3.

Relaxation rates, order parameters, and corresponding errors were then given to pyDR (https://github.com/alsinmr/pyDR), and analyzed using a total of 5 automatically optimized detector windows.

### Protein and Lipid Quantification

MLV samples were resuspended in pH 4 buffer (50 mM NaAcetate, 150 mM NaCl), as appropriate. For complete solubilization, aliquots were mixed 1:1 (v/v) with MLV resolving buffer (50 mM Tris, 150 mM NaCl, 50 mM SDS, pH 7). The concentrations of GlpG protein were determined by absorption measurements at 280 nm at a Nanodrop 1000 spectrometer (NanoDrop Technologies, Wilmington, DE, USA). The concentration of the LacYTM2­(EDANS/DABCYL) kinetic substrate was determined by measuring the absorbance of the DABCYL moiety at 453 nm. Absolute peptide concentrations were calculated from a linear calibration curve generated using LacYTM2­(EDANS/DABCYL) dissolved in DMSO.

MLV samples were resuspended in pH 4 buffer (50 mM NaAcetate, 150 mM NaCl). Quantitative lipid extraction was performed according to the Bligh and Dyer method.^31^ Briefly, 100 μL of buffer-solubilized MLV suspension was mixed with 200 μL CHCl_3_/MeOH (1:1, v/v) and vortexed for 30 s. Phase separation was achieved by centrifugation (5 min, 10,000 × g, 20 °C), and the chloroform phase containing the lipids was carefully collected and transferred into a new glass vial. To maximize recovery, the extraction was repeated twice with 100 μL CHCl_3_ per step, and the organic phases were combined. Solvent was removed under vacuum using an RVC 2–25 CDplus centrifugal evaporator (Martin Christ, Osterode, Germany). The resulting lipid film was stored at −20 °C until analysis by HPTLC.

Lipid concentrations were determined by high-performance thin-layer chromatography (HPTLC) followed by densitometric analysis of the resolved and visualized lipid bands. For quantitative analysis, dried lipid extracts were redissolved in CHCl_3_ and applied to HPTLC silica gel 60 plates (Merck KGaA, Darmstadt, Germany) by a CAMAG Linomat 5 sample applicator (CAMAG, Berlin, Germany). Calibration standards were spotted on the same plate to enable quantification. Chromatographic separation was performed in a glass development chamber (CAMAG) using a mobile phase consisting of chloroform/ethanol/water/triethylamine (30:35:7:35, v/v/v/v) for 45 min to resolve PE/PG/CL lipid. Lipid bands were visualized by primuline staining (100 mg primuline dissolved in 200 mL acetone/H_2_O, 4:1, v/v; Sigma-Aldrich, Taufkirchen, Germany) followed by UV illumination. Lipid concentrations in the MLVs were determined by densitometric analysis using a nonlinear calibration curve generated from the corresponding lipid standards. PG and CL were eluted at the same spot, and therefore, a PE/CL to GlpG ratio was calculated instead of a lipid:GlpG ratio.

### GlpG Functionality Assays in Membranes

For functional analyses,^32^ GlpG and the substrate LacYTM2­(EDANS/DABCYL) were coreconstituted into MLV at a molar ratio of 1:1.2 GlpG: substrate at pH 4 to suppress protease activity during sample preparation.^33^ Fluorescence-based activity measurements were performed at a final GlpG concentration of 6 μM in a total reaction volume of 50 μL at pH 7. The corresponding amount of GlpG MLV was incubated with 1 μL of 1 M Hepes (pH 7) in assay buffer (20 mM Hepes, 150 mM NaCl, pH 7). As negative control, the 6 μM GlpG was kept in pH 4 condition (50 mM NaAcetate, 150 mM NaCl, pH 4). Substrate turnover was monitored by recording EDANS fluorescence over time using an Infinite M200 plate reader (Tecan, Männedorf, Switzerland). Kinetic traces were analyzed in R (R Foundation for Statistical Computing, Vienna, Austria) and fitted to a monoexponential: *I*(*t*) = *I*_max_ – *I*_0_ × exp­(−*t*/τ) where *I*(*t*) represents the EDANS fluorescence intensity at time point *t*, *I*_0_ is the initial fluorescence (offset), *I*_max_ the maximal fluorescence signal, and τ the characteristic time constant describing the cleavage reaction.

## Results

We first investigated how the presence of CL influences the catalytic cycle of GlpG using a functionality assay developed by Strisovsky et al.^32^ The GlpG substrate LacYTM2 was conjugated with self-quenching fluorophores EDANS and DABCYL on either side of the cleavage site (Ser_11_ of LacYTM2) and coreconstituted^34^ with GlpG in membranes with increasing CL content at pH 4, where the protease is inactive because of protonation of His254.^33^ Rapid pH shift to 7.4 activates GlpG and initiates proteolysis. Subsequently, the EDANS labeled N-terminus of the cleaved LacYTM2 sheds from the membrane monitored by an exponential increase of EDANS fluorescence. The observed increase in fluorescence intensity was fitted with a monoexponential saturation curve providing a characteristic time constant (τ) describing the velocity of the cleavage reaction.^17^ Figure 1B shows a plot of τ as a function of CL concentration in POPE/POPG membranes (75/25 molar ratio) showing a decrease in the characteristic cleavage time of LacYTM2 by GlpG in CL-containing membranes. All CL concentrations investigated show an increase in cleavage velocity of about 40% compared to POPE/POPG membranes in the absence of CL. As the natural CL content of *E. coli* membranes is reported to be about 5–10%,^18,35^ all subsequent experiments were carried out in membranes of a composition of POPE/POPG/CL of 67.5/22.5/10 molar ratio.

Next, we looked at the impact of GlpG on lipid headgroup structure and dynamics using ^31^P NMR. As pioneered by J. Seelig et al.,^36^ the anisotropy of the ^31^P chemical shift represents a sensitive indicator for lipid headgroup interactions within membranes. In Figure 1C and D, ^31^P NMR spectra of the POPE/POPG membranes without or with 10 mol % CL in the absence and in the presence of GlpG are shown. POPE/POPG membranes in the absence of GlpG display the characteristic ^31^P NMR spectrum of membranes in a liquid-crystalline phase undergoing fast axially symmetric rotational diffusion about the long axis. Lineshape calculations describe the spectra using the sum of two anisotropic lines with Δσ_1_ = 40 ppm (90%) and Δσ_2_ = 45 ppm (10%) for POPE and POPG, respectively. In the presence of GlpG, a drastic change in line shape is observed, indicating the strong impact of GlpG on the headgroup orientation and mobility of the lipids. The spectra are difficult to calculate and the line shape deconvolution is not unequivocal. The calculation shown in Figure 1C was done using three anisotropic lines (Δσ_1_ = 28.5 ppm (34%), Δσ_2_ = 22.5 ppm (34%), and Δσ_3_ = 13.5 ppm (28%)) along with an isotropic contribution of 4% (supplementary Figure S3).

In the presence of 10 mol % CL (Figure 1D), the ^31^P NMR spectrum of the membrane in the absence of GlpG is characterized by shorter *T*_2_ relaxation times, which leads to broadening of the individual components of the powder pattern at comparable CSA indicating slower lipid motions in this ternary mixture. The line shape can be explained using an axially symmetric powder pattern with Δσ = 40 ppm and an isotropic line that contributes about 7% to the spectral intensity. In the presence of GlpG, again a drastic change of the ^31^P NMR spectrum is observed. The width of the spectrum is strongly reduced and the spectral line shape cannot be explained using a single chemical shift anisotropy. These features clearly indicate a strong perturbation of the lipid headgroup structure due to the interaction with GlpG. But no indication for inverse hexagonal phase formation is observed in these powder patterns in spite of the high content of lipids with negative intrinsic curvature. In an attempt to calculate the spectral line shape, we used three axially symmetric powder pattern with Δσ_1_ = 33 ppm (32%), Δσ_2_ = 24 ppm (25%), and Δσ_3_ = 13.5 ppm (35%) along with an isotropic signal of 8% (supplementary Figure S3).

From these results, we wanted to investigate the specificity of GlpG interactions with the individual lipid species. To this end, we prepared membranes of the same mixture but using *sn*-1 chain perdeuterated POPE-*d*_31_ and uniformly ^13^C-labeled CL (U–^13^C-CL). These stable isotopes allow for selective NMR measurements of the individual response of either POPE or CL in the mixture to the presence of GlpG. Supplementary Figure S4 shows the ^2^H NMR spectra and Figure 2A the order parameters of POPE-*d*_31_ in POPE/POPG/CL (molar ratio 67.5/22.5/10) mixtures in the absence and in the presence of GlpG. Clearly, a strong decrease in lipid chain order is observed when GlpG is incorporated in these membranes. From the order parameters, the average chain length can be calculated,^27^ plotted in the inset of panel A. A very large decrease in chain length from 14.3 Å to 12.1 Å is induced by GlpG. In the presence of CL, the effect of GlpG on the POPE order parameters is much attenuated. In the absence of GlpG, POPE order parameters are lower in the middle chain region compared to the membrane without CL. In the presence of GlpG, the POPE order parameter profile features lower order parameters in the middle chain again, which increases toward the C-2 segment. From these data, the chain lengths of CL-containing membranes were calculated, amounting to 13.8 Å in the absence and 13.5 Å in the presence of GlpG. This means that the GlpG-induced thinning of the membranes without CL that is induced by GlpG is reduced from 2.2 Å to only 0.3 Å in the presence of CL.

**Figure 2:**
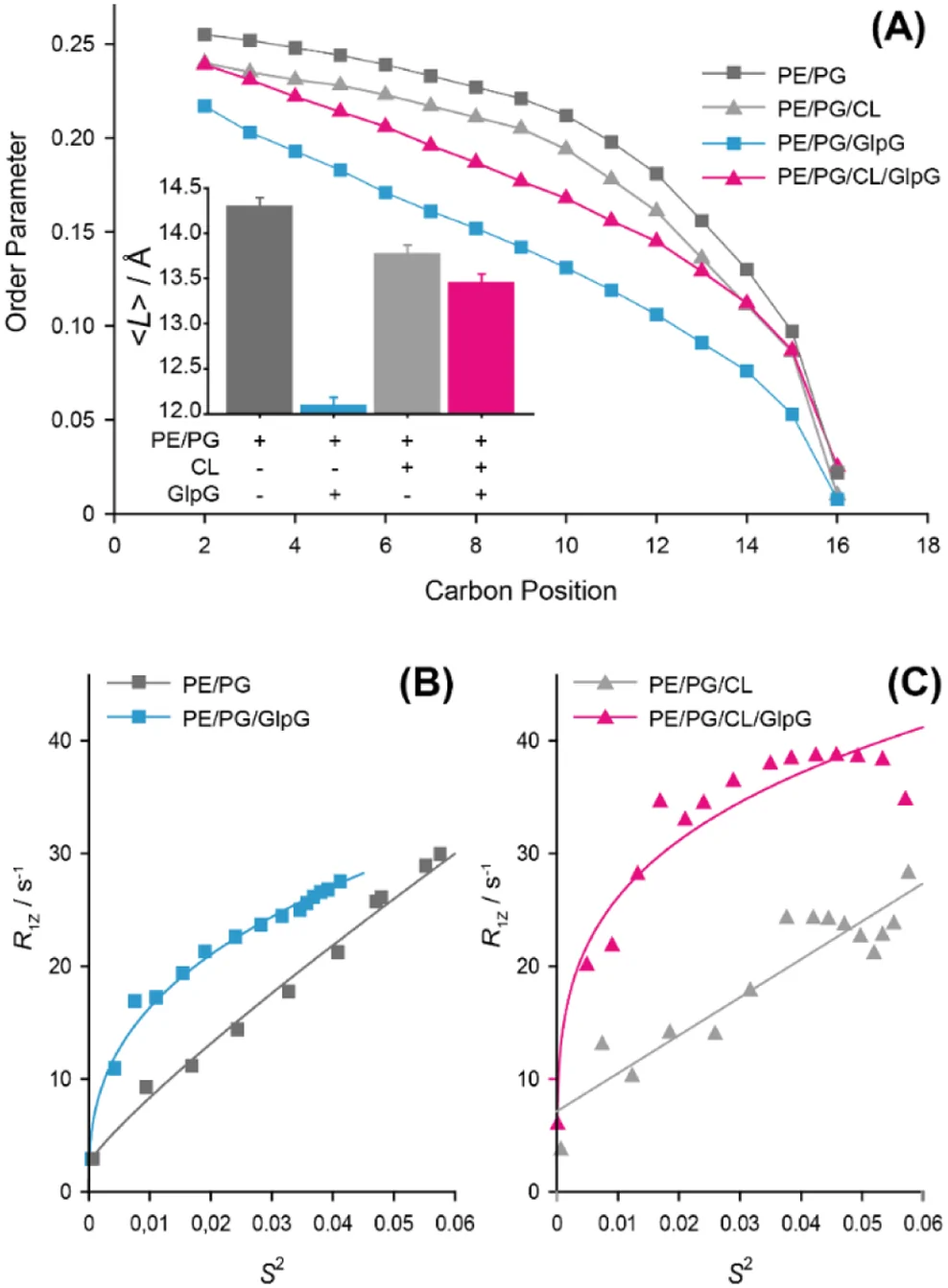
^2^H NMR data on the structure and dynamics of POPE/POPG membranes in the presence and in the absence of CL and GlpG (lipid to protein ratio 125:1). (A) ^2^H NMR order parameter plots of POPE-*d*_31_ in POPE/POPG mixtures (molar ratio 75/25) with and without CL in the presence (color) and in the absence of GlpG (gray). (B) correlation plot of the *R*_1Z_ vs *S* ^2^ for POPE-*d*_31_ in samples of POPE/POPG in the absence and in the presence GlpG. (C) correlation plot of the *R*_1Z_ vs *S* ^2^ for POPE-*d*_31_ in POPE-*d*_31_/POPG/CL mixtures (molar ratio 67.5/22.5/10) in the absence and in the presence of GlpG.

The strong difference in the response of the order parameters of POPE to GlpG in the absence and in the presence of CL suggests alterations in the interaction strength with the protein. To correlate this notion with the molecular dynamics of the lipid acyl chains, we carried out ^2^H NMR Zeeman order relaxation measurements. Experimentally determined ^2^H NMR *R*_1Z_ relaxation rates plotted against the square of the chain order parameter of each deuterated segment in the chain provides information on membrane elastic properties and the underlying lipid dynamics. As described by M. F. Brown and coworkers,^28,37,38^ in saturated lipid membranes, a plot of *R*_1Z_ vs *S* ^2^ provides a straight line, which serves as a reference for the liquid-crystalline phase state. In more rigid membranes, for instance induced by cholesterol, the *R*_1Z_ vs *S* ^2^ plot is also linear, but the slope is shallower. When detergents are added, which make membranes softer, the *R*_1Z_ vs *S* ^2^ plot shows curvature and a steeper initial slope.^28,37,38^

In Figure 2B and C, we plot *R*_1Z_ vs *S* ^2^ for POPE-*d*_31_ in samples of POPE/POPG in the absence and in the presence of CL and GlpG. Figure 2B compares the impact of GlpG on membrane elasticity for POPE/POPG membranes in the absence of CL. Without GlpG, membranes show typical square law plots for soft monounsaturated lipid membranes,^39^ while addition of GlpG increases their elasticity. In the presence of CL and GlpG (Figure 2C), we observe a strong increase in *R*_1Z_ relaxation rates, which is indicative of a pronounced softening of the membranes due to the presence of GlpG.

Considering the interesting modulation of membrane response to GlpG by CL, we wanted to study the structure and dynamics of the CL lipid component in the mixture. No isotopically labeled CL molecules are commercially available. Natural abundance ^13^C NMR measurements would suffer from signal superposition with the much higher concentrations of POPE and POPG in the mixture, rendering an unequivocal characterization of CL impossible. Therefore, we used U–^13^C-labeled CL, for which isotope enrichment of CL with ^13^C was achieved from an *E. coli* culture fed with U–^13^C glucose. Expression and U–^13^C-CL purification was carried out as described in the literature.^22^ With this molecule in hand, we have a tool for studying the structure and dynamics of CL in the mixture and the impact of GlpG on this specific lipid.

In Figure 3A, B, the ^13^C magic angle spinning (MAS) NMR spectra of U–^13^C-CL in the POPE/POPG/CL mixture in the absence and in the presence of GlpG are shown. Lipid signals are well resolved and assigned to the respective segments of CL. There are only a few very minor chemical shift changes in the presence of GlpG (Δδ < 0.2 ppm), which are below the digital resolution (∼0.3 ppm) and therefore not significant. The NMR spectrum shows prominent signals from the U–^13^C-labeled CL, but also some weak signal possibly of the PE headgroup at ∼42 (β) and 63 ppm (α). We used HPTLC to check if this represents an impurity in the U–^13^C-CL, but no contamination was found (supplementary Figure S5). Previous research also indicated that some PE and PG remains bound to GlpG after purification.^17^ This amounts to only 5% of the total lipid at natural abundance ^13^C and cannot account for these signals. Possibly, these could represent the ^13^C natural abundance signal from the POPE in the sample (67.5% of the total lipid). Alternatively, these signals could also be due to somewhat “unusual” lipid chains produced in bacteria.^40^ For instance, the signals at ^13^C chemical shifts of 12 and 17 ppm are assigned to cyclopropane fatty acids of CL as ESI-MS analysis showed CLs with odd carbon chains (C65, C67, C69); see supplementary Figure S1 and Table S4. Other fatty acid modifications of bacterial systems contain cyclohexyl rings,^40^ which resonate between 26 and 43 ppm.

**Figure 3:**
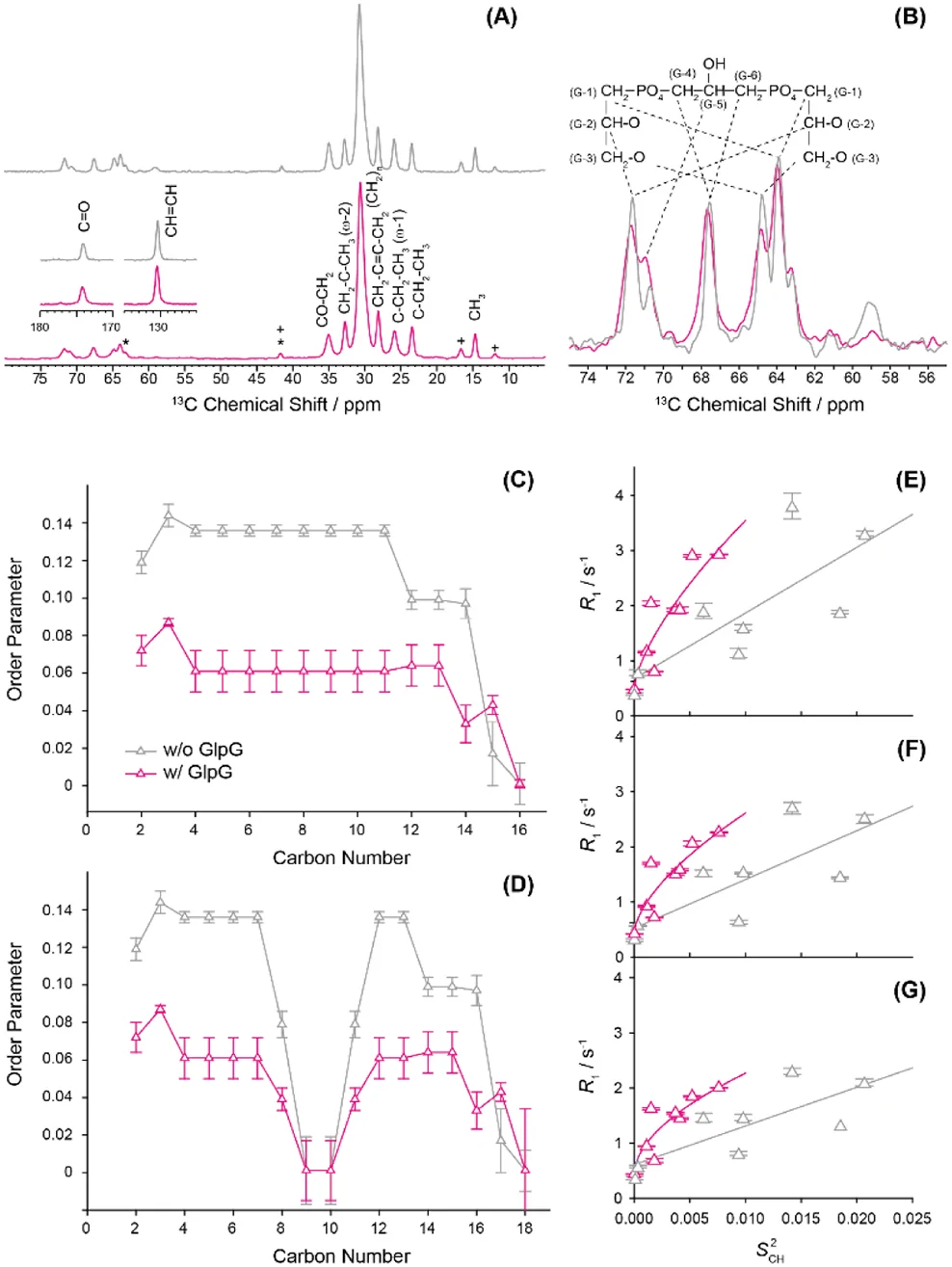
^13^C MAS NMR spectra, order parameter, and relaxation times of U–^13^C labeled CL in mixtures of POPE/POPG/CL (molar ratio 67.5/22.5/10) in the absence (gray) and in the presence of GlpG (magenta). (A) and (B) show the ^1^H-decoupled and directly excited ^13^C MAS NMR spectra with assignments. Signals labeled with * and + indicate signals that possibly arise from the PE headgroup (at natural abundance) and specific fatty acids found in bacteria.^40^ (C, D) ^1^H–^13^C NMR order parameters of the saturated (C) and unsaturated (D) chains of U–^13^C CL in POPE/POPG/CL membranes the absence (gray) and in the presence (magenta) of GlpG. Note that panels (C) and (D) repeat the same data as at the given experimental resolution, there is no distinction between *sn*-1 and *sn*-2 chain signals. The only unique *sn*-2 chain signals come from the double bond carbons. (E–G) correlation plots of ^13^C *R*_1_ vs *S*_CH_^2^ for U–^13^C CL in POPE/POPG/CL membranes the absence (gray) and in the presence (magenta) of GlpG at *B*_0_ field strengths of 16.4 T (E), 14.1 T (F), and 9.4 T (G).

Next, we measured *T*_1_ relaxation times at three magnetic field strengths (16.4, 14.1, and 9.4 T) and ^1^H–^13^C dipolar couplings using DipShift^41^ experiments (NMR data shown in the Supporting Information, selected fits in Figures S6 – S8, rate constants in Tables S2-3).

First, we analyzed the ^1^H–^13^C dipolar couplings of the CL acyl chain by calculating the ^1^H–^13^C order parameters. As with the ^2^H NMR order parameters, which report the amplitude of reorientation of the ^2^H–C bond vector, also the ^1^H–^13^C order parameters provide information on the dynamics of this bond vector. While the interaction of the quadrupolar moment of ^2^H with the electric field gradient over the ^2^H–C bond is probed in ^2^H NMR, the ^1^H–^13^C dipolar coupling shows the same axially symmetric geometry along the ^1^H–^13^C bond, providing the same order parameter. However, the time scale of motions to which the experiment is sensitive is slightly different as the quadrupolar coupling constant in a ^2^H–C bond is 167 kHz while the ^1^H–^13^C dipolar coupling is only 23 kHz. This means that motions with correlation times <6 μs contribute to *S*_CD_, while also slower motions (τ < 44 μs) lead to averaging of the ^1^H–^13^C dipolar coupling.

Figure 3C, D shows the ^1^H–^13^C order parameters of CL in the lipid mixture in the absence and presence of GlpG. As there is no complete site resolution for all chain methylene signals in the NMR spectrum, a plateau value is reported for the upper/middle chain.^42,43^ However, resolution allows reporting order parameters for the saturated (Figure 3C) and the unsaturated (Figure 3D) chains of CL individually. In the latter, the order parameters of the carbon segments next to the double bond are very low in agreement with previous reports.^42,43^ The order parameters of CL in the absence and presence of GlpG are strikingly different with much lower order parameters in the presence of GlpG. As discussed before, chain order decrease is associated with a decrease in the average chain length. Employing the same mean-torque potential model as for the ^2^H NMR data,^27^ we find a decrease in the CL lipid chain length of Δ*L* = 2.2 Å induced by GlpG. Given the strong impact of GlpG on CL chain order parameters, we wanted to investigate in detail how GlpG influences the motions of these chains. To this end, we plotted the ^13^C *R*_1_ relaxation rates of all lipid segments as a function of the square of the order parameter for each chain segment (Figure 3E–G). Similar to the ^2^H NMR data of POPE in the mixture (cf. Figure 2B, C), the presence of GlpG leads to plots that show a much softer membrane and a more mobile CL component.^44^

^13^C NMR relaxation rates contain a wealth of dynamics information allowing to precisely describe the dynamic landscape of the membrane over a correlation time window form 10s of picoseconds to 100 s of microseconds.^45^ In addition to the *T*_1_ relaxation and dipolar coupling data, we also measured *T*_1ρ_ relaxation data at spin lock fields of ∼8, 12, and 22 kHz and heteronuclear ^1^H–^13^C NOE values (at 600 and 700 MHz). With this comprehensive data set, we calculated the detector response^45,46^ for the CL molecules and compare membranes in the absence and in the presence of GlpG (Figure 4). The amplitude of the detector response provides the contribution of the motions at this particular time scale.^46^ A plot with the correlation time sensitivities of these detectors is shown in Figure 4A covering lipid motions from ∼100 ps to ∼100 μs.

**Figure 4:**
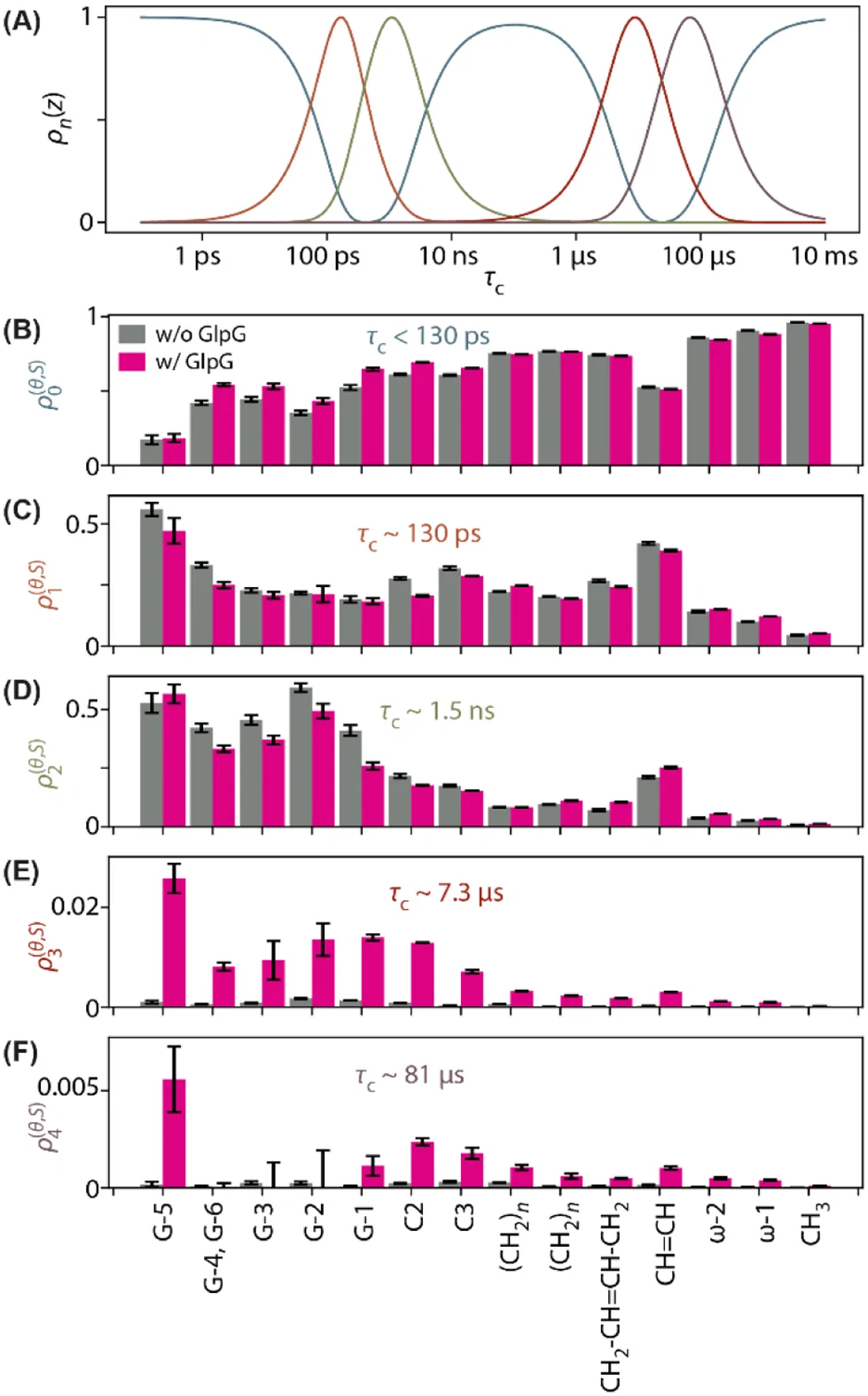
NMR detector response of ^13^C-labeled CL in membrane mixtures of POPE-*d*_31_/POPG/U–^13^C-CL mixtures (molar ratio 67.5/22.5/10) in the absence and presence of GlpG. Panel (A) shows the construction of detector windows from the *T*_1_ relaxation times at three Larmor frequencies, *T*_1ρ_ relaxation times at spin lock fields of 8, 12, and 22 kHz, hetNOE data, and motionally averaged ^1^H–^13^C dipolar couplings. Panels (B–F) displays the individual detector responses for all lipid segments with resolved NMR signals in a given correlation time window.

Figure 4B–F compares the individual detector response for each lipid segment of CL in the lipid mix for the absence and presence of GlpG. There are no remarkable differences observed for the fast motions with correlation times up to ∼1 ns (detectors ρ_0_ to ρ_2_, Figure 4B–D). However, a drastic increase in the populations of slower motions with correlation times in the microsecond range is observed (detectors ρ_3_ and ρ_4_, Figure 4E, F). In the absence of GlpG, these slow motions do not contribute much to the mobility of CL, however, the slow CL motions are much more relevant in the presence of GlpG.

## Discussion

The question how lipids influence membrane protein function remains largely unanswered as the overwhelming multitude of lipid species in the plasma membrane as well as lateral and transversal heterogeneity are difficult to represent in biophysical systems.^6^ There is consensus that specific protein–lipid interactions can have a profound impact on protein function. Lipids like PIP_2_, Gb3, or PS are active modulators in protein function.^14,47–50^ But also the bulk properties like curvature, membrane thickness, electrostatics, or elastic properties have a profound impact on protein function.^5–7,11,16,17,51–54^ While spectroscopic tools provide the means to address such questions, difficulty arises because lipid structures are quite similar as they often only vary in their headgroup. Therefore, unequivocal detection of a unique molecule by spectroscopic tools suffers from signal superposition and dynamic range problems of the receivers especially when it only constitutes a small fraction of all lipids. Here, we use a reconstituted system of three lipid species and the small rhomboid GlpG to probe the impact of a small concentration of CL on membrane structure and protein function exploiting isotope effects.

Several studies have reported the importance of the lipid environment on GlpG function.^16,17,55–57^ GlpG thins the membrane in some systems,^16,17,58^ which influences protein function suggesting an allosteric effect of the membrane environment on embedded GlpG. We could detect thinning only of POPE/POPG membranes, but not in other PC or POPC/POPG bilayers.^16,17^ Due to the high negative intrinsic curvature of POPE, thinning of POPE-containing membranes is required for protein function suggesting that hydrophobic matching between protein and membrane is the most important parameter in GlpG function.

The current results indicate that CL is an essential lipid for GlpG function. First, in the presence of CL, the characteristic time for substrate cleavage is decreased by ∼40% in a concentration independent manner. Second, drastic line shape alterations in ^31^P NMR spectra of the membrane occurred but are not specifically assignable to CL. Third, significant order parameter alterations for the CL in the presence and absence of GlpG were observed (cf. Figures 2A and 3C, D).

Although it was reported that CL stiffens membranes,^19^ the POPE order parameters were slightly lower when CL is present (Figure 2A), indicating that CL increases the fluidity of the membrane (Figure 2A). While the presence of GlpG decreases the POPE chain length by 2.2 Å in the absence of CL, in the presence of 10 mol % CL, this reduction is much attenuated and amounts to only 0.3 Å.

The obvious question to ask is whether or not GlpG has a similar impact on CL chain length as it had on POPE chain length in the absence of CL. As no ^2^H-labeled CL is commercially available, we purified CL from an *E. coli* culture that was fed with U–^13^C glucose as precursor, yielding U–^13^C-labeled CL.^22^ With this lipid in hand, acyl chain order parameters could be calculated from the motionally averaged ^1^H–^13^C dipolar couplings measured in a ^13^C-detected MAS separated local field experiment. It is seen that in the presence of GlpG, the chain order parameters of CL are much reduced both in the saturated and unsaturated acyl chains. When calculating the acyl chain lengths, we remarkably see a 2.2 Å reduction of the CL chains induced by GlpG. Before interpreting this result, we needed to analyze the acyl chain composition of CL by mass spectrometry (supplementary Figure S1 and Table S4).

About half of the CL molecules featured acyl chains comprising in total 66 to 70 carbons with mostly two or three double bonds in agreement with previous gas chromatographic analysis.^22^ Some of the most prominent CL species also contain odd-numbered acyl chains, which could be confirmed by MS/MS experiments.^59,60^ Although the presence of odd-carbon number acyl chains either containing a double bond or cyclopropyl group could not be determined with the used methods, the effect on GlpG should be similar. Also, the impact of CL 67:2 and CL 68:2 as the two most abundant species should be nearly the same since only one acyl chain shifted from fatty acid 18:1 in CL 68:2 to 17:1 (or 17-cyclo) in CL 67:2.

After confirming that the natural CL species contain mostly palmitoyl and oleoyl chains, we plot the order parameters of CL in Figure 3 and compare the absence and presence of GlpG. This puts us in the position to hypothesize why GlpG reduces the chain length of POPE in the absence of CL but not in its presence. The fact that in the presence of GlpG, CL exhibits significant disordering along with a strong reduction of its chain lengths can be explained by a restructuring of the CL-containing membrane by the presence of the protein. In the absence of CL, the protein–lipid interface is dominated by POPE-GlpG contacts. Due to the hydrophobic mismatch between the thickness of GlpG and the chain length of POPE, the chains of the lipid become disordered (i.e., shorter) to match the smaller hydrophobic thickness of the protein.^17^ In the presence of CL, we propose that the POPE at the interface with the protein is replaced by CL. CL then preferentially interacts with GlpG requiring a chain length reduction for hydrophobic matching. The POPE formerly associated with GlpG in the absence of CL are released into the bulk where they are less influenced by the protein and assume a less perturbed structure expressed by higher chain order.

The putative restructuring of the membrane also emphasizes its heterogeneity, which clearly manifests in the different contributions and broadening of the ^31^P NMR spectra (supplementary Figure S3), which are sensitive to a time scale of ∼100 μs indicating slow to intermediate time scale exchange of lipids. The ^2^H NMR spectra, which are sensitive on a faster time scale of <10 μs show broadening but there is only one population detected in support of intermediate to fast exchange (supplementary Figure S4). This points toward a transient heterogeneity of the lipid distribution in GlpG-containing membranes.

Such a lateral membrane restructuring should lead to alterations in lipids dynamics in particular for CL. Correlation plots of the segmental Zeeman order relaxation rate *R*_1_ vs the order parameter of the respective segment squared (*S*_CD_^2^) provide information on the membrane’s elastic properties.^37,61^ When plotting the ^2^H NMR *R*_1Z_ relaxation rates vs. the order parameters of POPE squared, we find that GlpG insertion leads to an increase in membrane elasticity (Figures 2B, C). The same is observed when analyzing the ^13^C relaxation rates and order parameters of CL (Figure 3C–E). While both measurements report an increase in elasticity representing a membrane property on the mesoscopic length scale, it is not sensitive to the individual lipid component. We relate this increased membrane softness to the matching of membrane and GlpG’s hydrophobic thicknesses but do not get information on the interaction of GlpG with a specific lipid species.

To answer the question if there is a specific interaction of GlpG with CL, we also measured several ^13^C relaxation rate constants in the rotating frame under spin lock conditions, heteronuclear nuclear Overhauser effects, and ^1^H–^13^C dipolar couplings with site resolution over the individual segments of the lipid. Using detector analysis,^46^ we could quantify the relevance of a given motion within a specific correlation time window. When comparing CL-containing membranes in the absence and in the presence of GlpG, there is no large difference when we consider fast motions (Figure 4). These fast motions of CL with correlation times of several hundreds of picoseconds (represented by detectors ρ_0_ and ρ_1_) are segmental in nature^62^ and obviously not strongly influenced by interaction with GlpG. The next detector, ρ_2_, is sensitive to somewhat slower motions with correlation times around 1.5 ns and represent motions of entire lipids such as the axially symmetric rotational diffusion.^62^ For some segments (i.e., the headgroup and glycerol moieties) a small decrease in the detector response ρ_2_ is observed when GlpG is present, suggesting that this motion then has a smaller relevance.

But the most important change is observed in the detector responses ρ_3_ and ρ_4_, which are sensitive to correlation times of ∼6 μs (ρ_3_) and ∼60 μs (ρ_4_). These motions are almost nonexistent in the absence but highly relevant in the presence of GlpG as illustrated in Figure 5. Such slow lipid motions would typically reflect undulations of large membrane patches, which are not present in GlpG-containing membranes at the given conditions. It is worthwhile to consider the time scale of the GlpG motions in lipid membranes. Considering the Saffman–Delbrück theory^63^ one can estimate a rotational diffusion time of τ_R_ ∼20 μs for GlpG considering its dimensions from the X-ray structure^64^ and a membrane viscosity of 1 P. A τ_R_ of around 20 μs agrees the correlation time sensitivity of detectors ρ_3_ and ρ_4_ and suggest that at least part of the CL moves together with GlpG in the membrane implying relatively significant interaction with the protein. One would expect such a behavior for the annular lipids, which in this case are suggested to be mostly CL. However, CL most likely is not a structural or tightly bound lipid which would have led to ^13^C chemical shift changes, which were not observed. This interpretation would be in agreement with a recent coarse grained molecular dynamics simulation (CG-MD),^21^ which showed an extended residence time of CL of 170 ns at a predicted site of GlpG. A 1.4 kDa adduct was also found in native mass spectrometry, but it could not unequivocally be assigned to CL.^21^ In agreement with a preferential interaction of CL with GlpG, Lange and coworkers analyzed the lipidome of two *E. coli* strains and compared it to the lipids coextracted with GlpG by *n*-decyl β-maltoside (DM) or diisobutylene-maleic acid (DIBMA).^55^ Interestingly, they found an increased concentration of CL coextracted with GlpG compared to the lipidome of *E. coli*. This supports our finding that CL is more closely associated with GlpG in the membrane.

**Figure 5:**
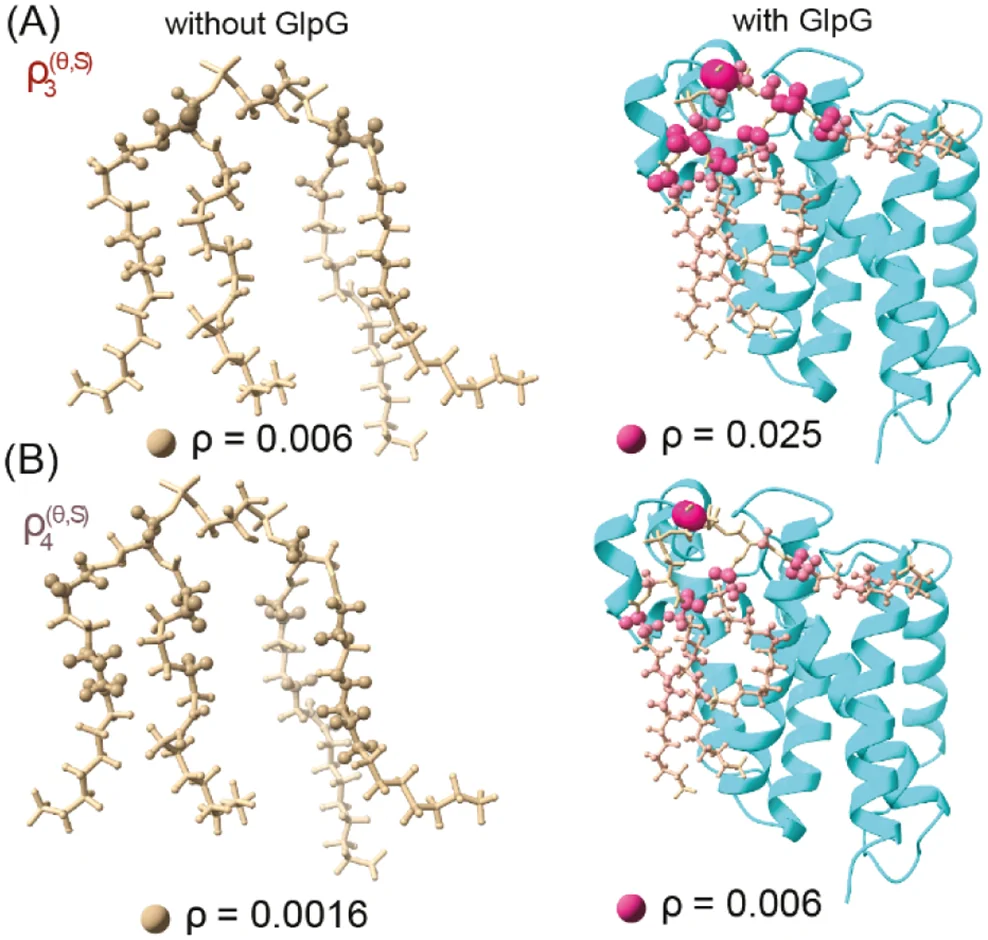
Illustration of the difference in the detector responses ρ_3_ (A) and ρ_4_ (B) of on the motions of CL. In the absence of GlpG (left), there are almost no slow motions in this correlation time window. In the presence of GlpG (right), these CL motions are clearly much more relevant agreeing with the rotational diffusional motions of GlpG.

The stoichiometry of the sample allows estimating the number of CL molecules that are found in the annulus around GlpG. At the lowest CL concentration (5 mol % CL), we have about 6 CL per protein. The lipid annulus around GlpG comprises about 14 lipids given the dimension of the molecule from the crystal structure.^64^ Even at this low CL concentration, the impact of the lipid in accelerating proteolysis reached its maximum, suggesting that modulation of protein function depends on ≤6 CL molecules. Likely, the other lipids are PE. At 10 mol %, where all NMR measurements of the current study were carried out, the lipid annulus of GlpG could theoretically consist of only CL. Nevertheless, some lipid exchange between the lipid annulus and the next lipid shell would be entropically favorable.

It may be perceived as a contradiction that we propose CL to be preferentially associated with GlpG but still exhibits high dynamics on the fast time scale as represented by detectors ρ_0_ and ρ_1_. One argument in favor of such a scenario is the aforementioned lipid exchange which increases the entropy of the system. While favorable free energy would result from most optimal electrostatic interactions and hydrogen bond formation between lipids and protein, highly immobilized lipids have a very unfavorable entropy, which is why often no or only few lipids are found in crystal or cryo-EM structures of membrane proteins. By fast *trans*-*gauche* isomerization along with shortening of the chain length of associated CL, part of the entropy loss is compensated by higher chain entropy. By retaining much of its dynamics, associated or annular lipids can still sample numerous microstates which keeps the entropy high and allows for lowering the lipid chain order parameters to adjust the membrane thickness in the lipid annulus.

In a previous study, GlpG was crystallized in a DMPC/CHAPSO environment and the crystal structure showed 14 fully or partially ordered DMPC molecules in the grooves and crevices of the protein.^65^ But no structure for GlpG crystallized from PE or CL is available. Coarse-grained molecular dynamics identified a putative CL binding site on GlpG, involving residues Arg_92_, Trp_98_, Tyr_160_, and Lys_167_.^21^ While the coarse-grained representation does not allow for a very detailed atomistic interpretation of this site, an increased residence time was found.

All NMR measurements were done at pH 4, where the protein is inactive. GlpG is prone to autocatalytic cleavage at neutral pH,^17^ which would become a problem over the long measuring times for collecting all NMR data for order parameter and detector analysis. In our previous work we compared membrane thinning induced by GlpG at pH 4 and pH 7 and found no differences.^17^ Although GlpG is inactive at low pH, the tertiary structure remains intact and so do the lipid–protein interactions. For the lipids, the p*K*_a_ of PE and PG is well below 4 and lipid charge should not be affected at pH 4.^66^ While CL protonation behavior has been more debated, two studies reported either a p*K*_a_ of 2.15 and 3.15 for the different phosphates^67^ and another study reported a p*K*_a_ of 1.0 and 1.59.^68^ Therefore, CL should have a charge of −2 at pH 4 and at neutral pH.

Finally, we wondered about the mechanism of the preferential interaction between CL and GlpG. The most obvious characteristics of CL is its high negative charge and the possibility to interact with positively charged side chains of GlpG. We had previously tested if the negatively charged POPG modulates the activity of GlpG and if GlpG influences the properties of POPC/POPG membranes.^17^ Neither was the case: there was no difference in the activity of GlpG and its impact on membranes in pure POPC and POPC/POPG bilayers.^17^ Therefore, we postulated specific PE-GlpG interactions, induced by the high negative intrinsic curvature of PE. There is of course the possibility that CL could interact more strongly because of its higher negative charge.^21^ Yang and coworkers indeed suggested that the binding mode of CL to a membrane protein is different than PG.^69^ Thus, the doubly charged CL could outcompete monovalent PG, as shown in the CG-MD simulation.^21^ While charge effects should be relatively well represented in CG-MD, the lack of full atomic structure may oversimplify other molecular features. An important parameter that POPE and CL share is their negative intrinsic curvature (*c*_0_ < 0). The intrinsic curvature for POPG is reported to be vanishing (*c*_0_ ∼ 0), highly negative values are reported for POPE (*c*_0_ = −0.3 nm^–1^) and significantly higher negative values are reported for CL (*c*_0_ = −1.1 nm^–1^).^70,71^ This suggests that in addition to the high negative charge density of CL, also the negative intrinsic curvature of CL represents an important molecular property responsible for the interaction with GlpG.

## Conclusions

Our study reveals a pivotal role of the lipid cardiolipin in the structure and lateral organization of *E. coli* membranes. It actively modulates GlpG function leading to faster proteolysis. Along with the high charge density of CL, GlpG function seems sensitive to membrane curvature influencing the lateral organization of the membrane and its entire dynamic landscape. On a more general level, the diversity of the lipidome of biological membranes, the dynamics, and the lateral and transversal heterogeneity of the lipid molecules provide regulatory and modulatory means to up- or downregulate protein function in a highly transient and dynamic fashion.

## Supplementary Material



## Data Availability

Code for analysis can be found at pyDR (https://github.com/alsinmr/pyDR).

## Notes

Figure S1: mass spectrum of purified CL fraction from *E. coli*, Figure S2: thin layer chromatography plate for the quantitative measurement of the lipid composition; Figure: S3 31P NMR spectra of POPE/POPG/CL in the absence and presence of GlpG; Figure S4: 2H NMR spectra of POPE-d31/POPG/CL in the absence and presence of GlpG; Figure S5: thin layer chromatography plate for checking the purity of the CL from *E. coli* culture; Figure S6: 13C NMR spectrum assignment and fit for relaxation data analysis, Figure S7: examples of the fitting curves of T1 relaxation data, Figure S8: examples of the fitting curves of T1ρ relaxation data; Tables S1: 2H NMR order parameters; Table S2: experimental parameters for the 13C MAS NMR investigations; Table S3: relaxation rates and standard deviations for the 13C MAS NMR investigations; Table S4: identified CL species using electrospray ionization mass spectrometry (PDF)
