# Supplementary material for: Cardiolipin Modulates the Activity of the Intramembrane Protease GlpG Inducing Membrane Restructuring
Source: J Am Chem Soc. 2026 Aug 27;148(35):38021–32. doi: 10.1021/jacs.6c09491 (PMC13564412; doi:10.1021/jacs.6c09491)
Supplement: Supplementary file 1 [file ja6c09491_si_001.pdf]

Supporting Information

Cardiolipin Modulates the Activity of the Intramembrane Protease GlpG Inducing Membrane Restructuring

Barbara Maier,[1,†] Oskar Engberg,[1,†] Viola Döbel,[1] Jenny Leopold,[1] Holger A. Scheidt,[1] Rosalie A. M. van Beekveld,[2] Markus Weingarth,[2] Albert A. Smith,[1] Daniel Huster[1,*]

[1] Institute of Medical Physics and Biophysics, Härtelstr. 16/18, 04107 Leipzig, Germany
[2] Universiteit Utrecht, NMR, Padualaan 8, 3584 CH Utrecht, The Netherlands

[†] these authors contributed equally

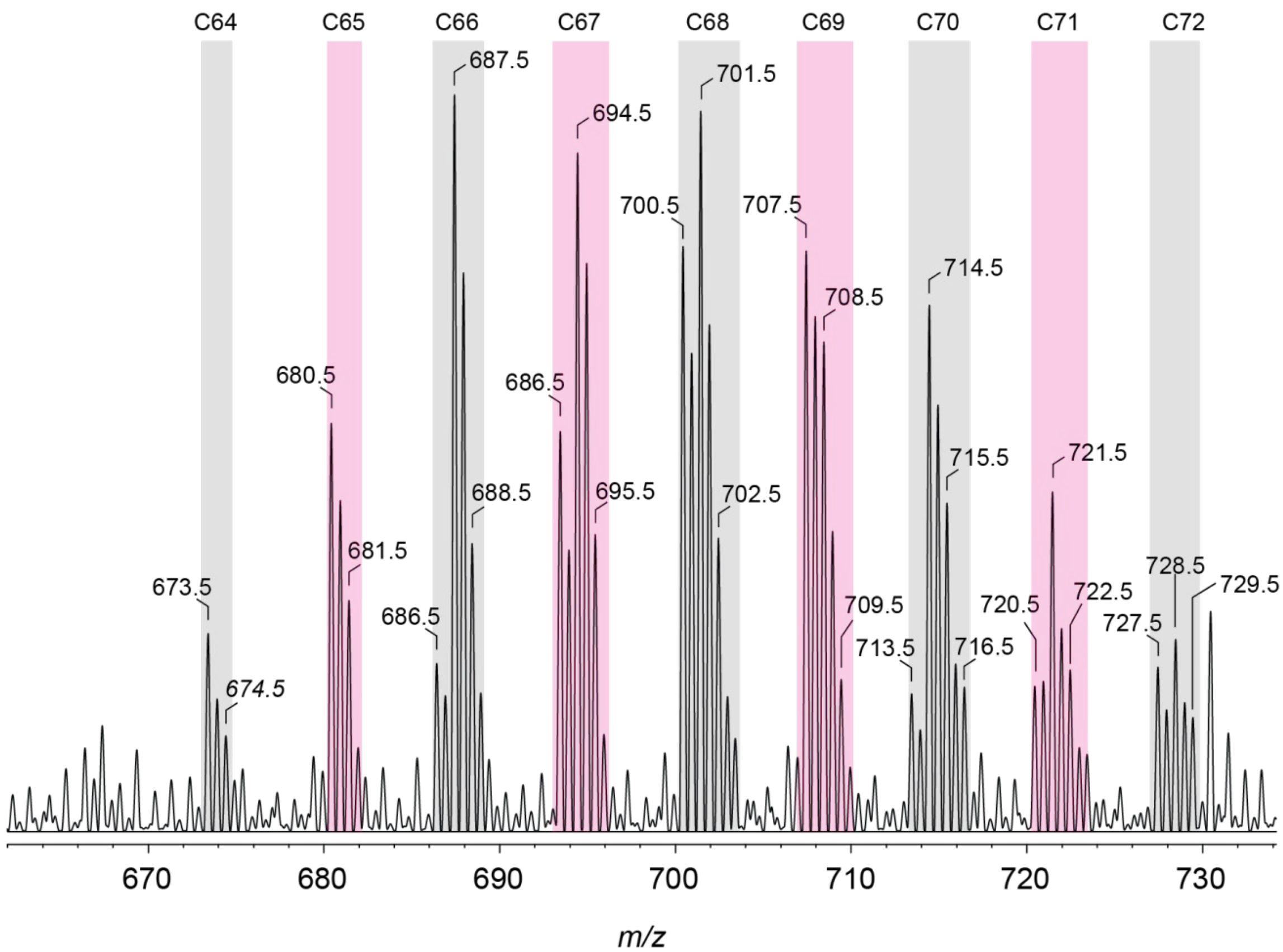

Fig. S1.

Mass spectrum of purified cardiolipin fraction from *E. coli* obtained by negative ionization electrospray ionization mass spectrometry (ESI MS) after direct infusion. The doubly negatively charged ions are represented as $[M-2H]^{2-}$, as these ions were generated preferentially over singly charged ions. The cardiolipin species assigned to the corresponding *m/z* values are summarized in Table S4.

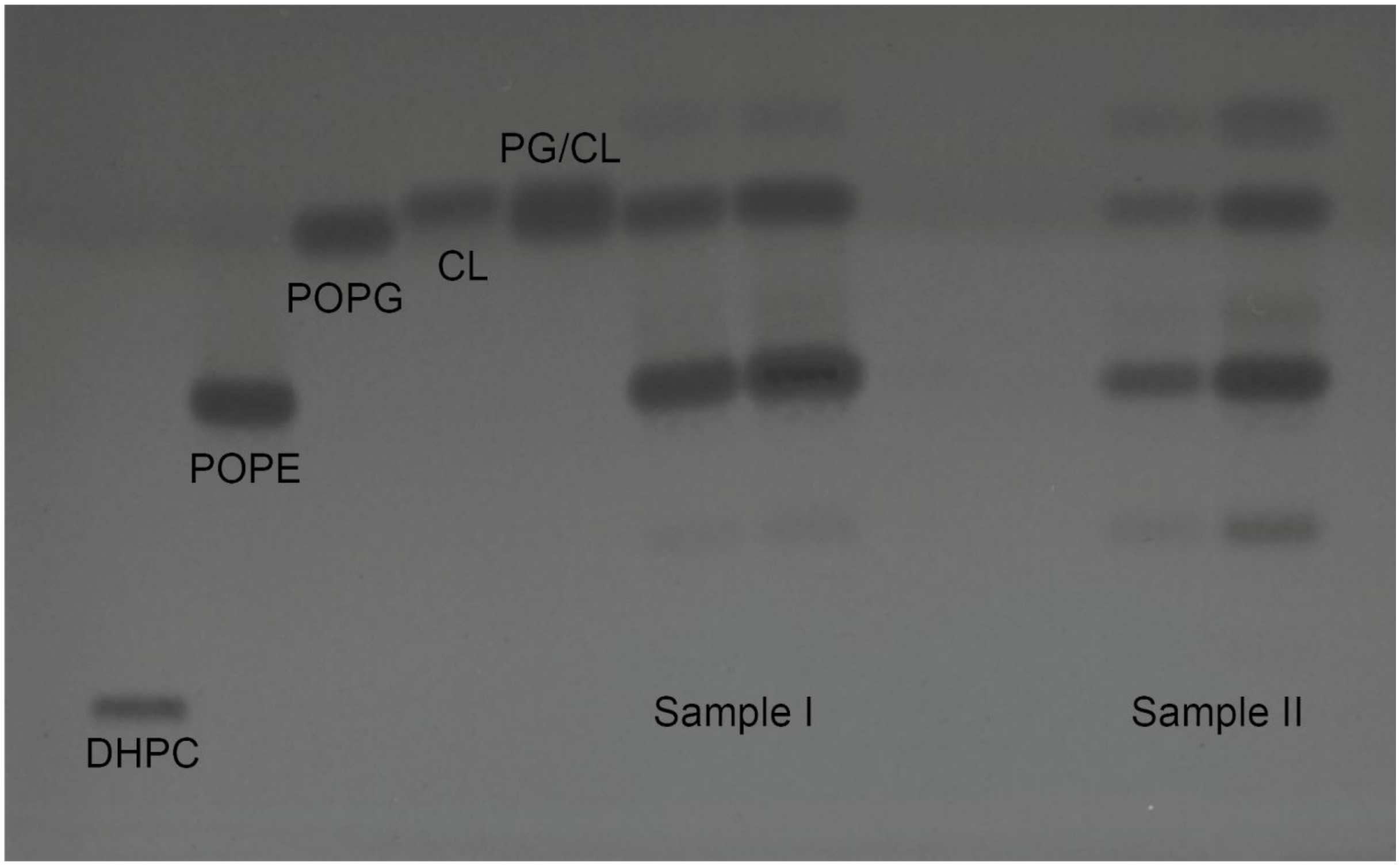

Fig. S2.

Thin layer chromatography plate for the quantitative measurement of the lipid ratios in typical samples investigated. Sample I: GlpG in U-^{13}C-CL/POPE/POPG MLV, Sample II: GlpG in POPE/POPG MLV.

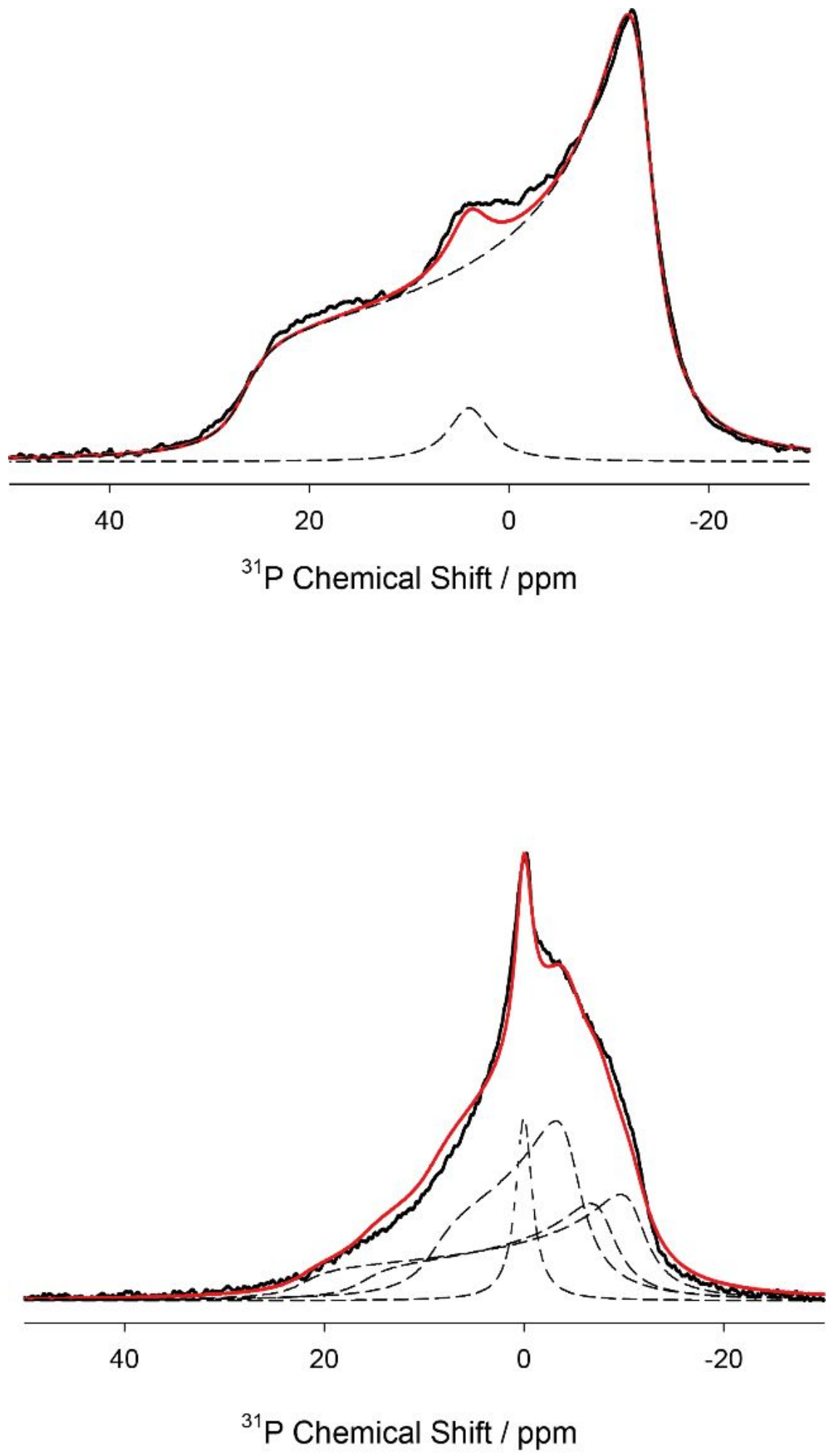

Fig. S3.

^{31}P NMR spectra of POPE/POPG/CL (molar ratio 67.5/22.5/10) in the absence (top) and in the presence of GlpG (bottom) at a prepared protein to lipid molar ratio of 1:125 and a temperature of 37°C. Red line represent best fit lineshape calculations and the dashed lines the individual powder spectra and isotropic lines necessary for fitting the overall lineshape.

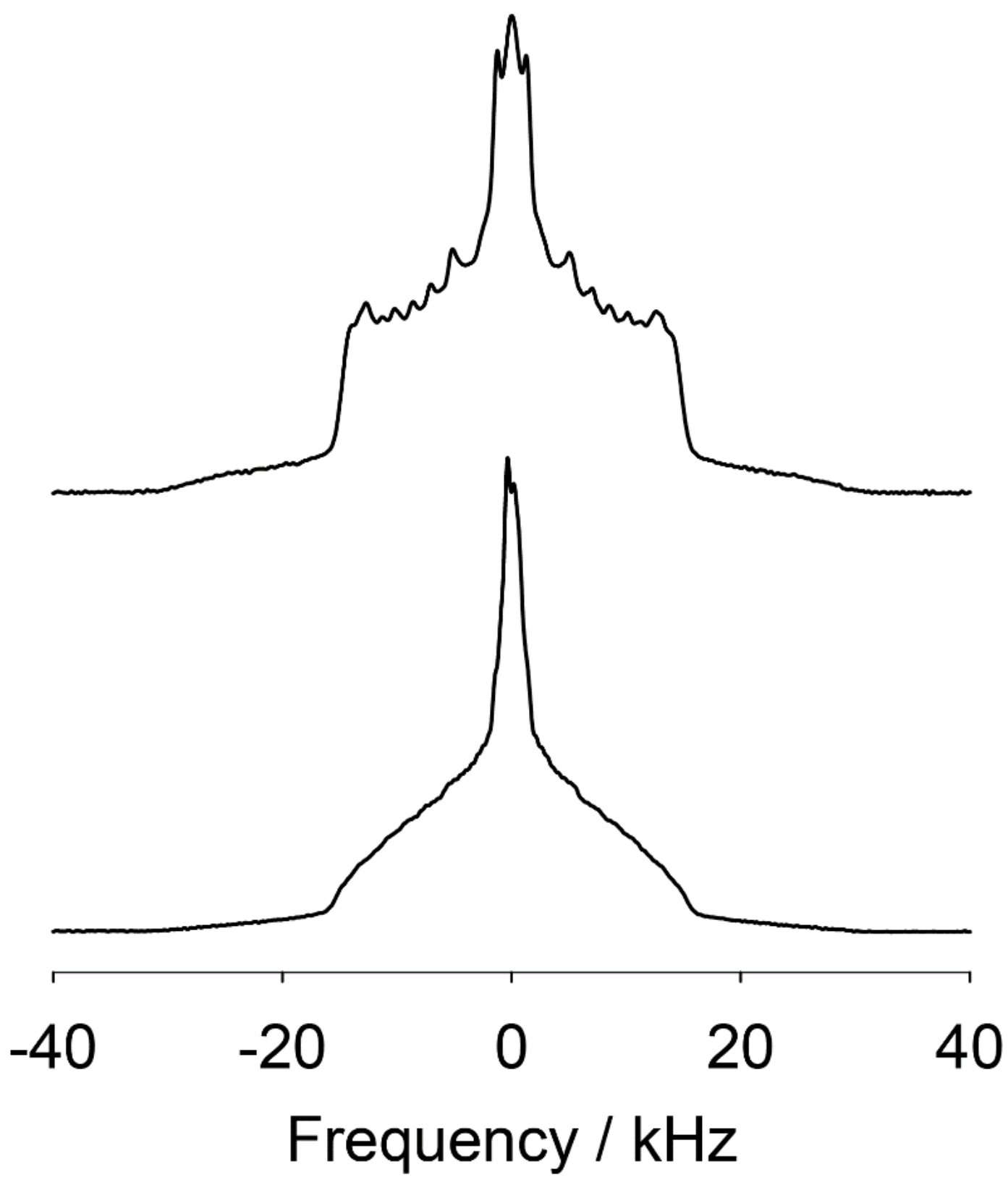

Fig. S4.
^{2}H NMR spectra of POPE-d_{31}/POPG/CL (molar ratio 67.5/22.5/10) in the absence (top) and in the presence of GlpG (bottom) at a prepared protein to lipid molar ratio of 1:125 and a temperature of 37°C.

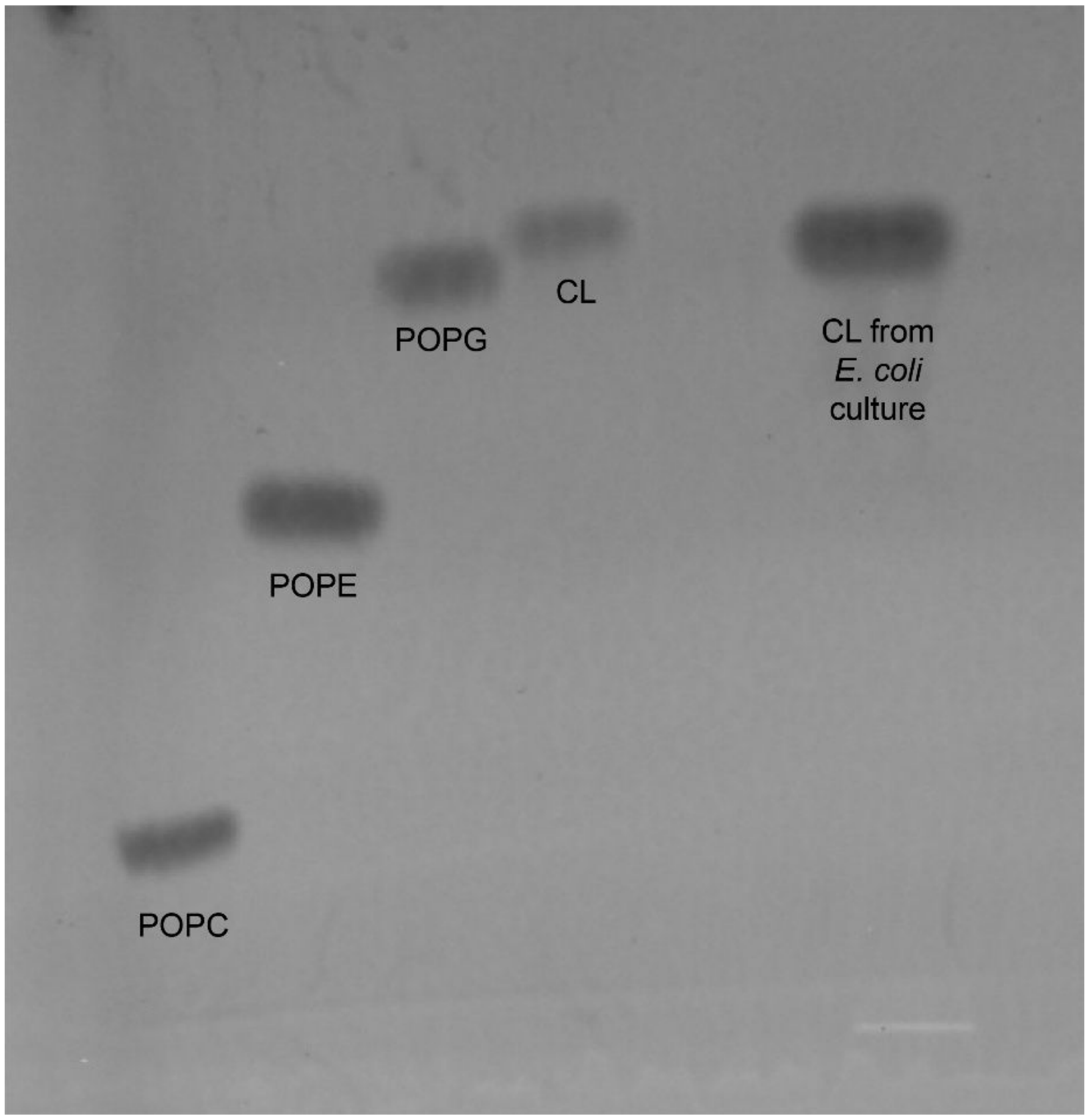

Fig S5.
Thin layer chromatography plate for checking the purity of the CL from *E. coli* culture. The left four lanes indicate the standard lipids and the right lane the CL confirming the absence of any impurities.

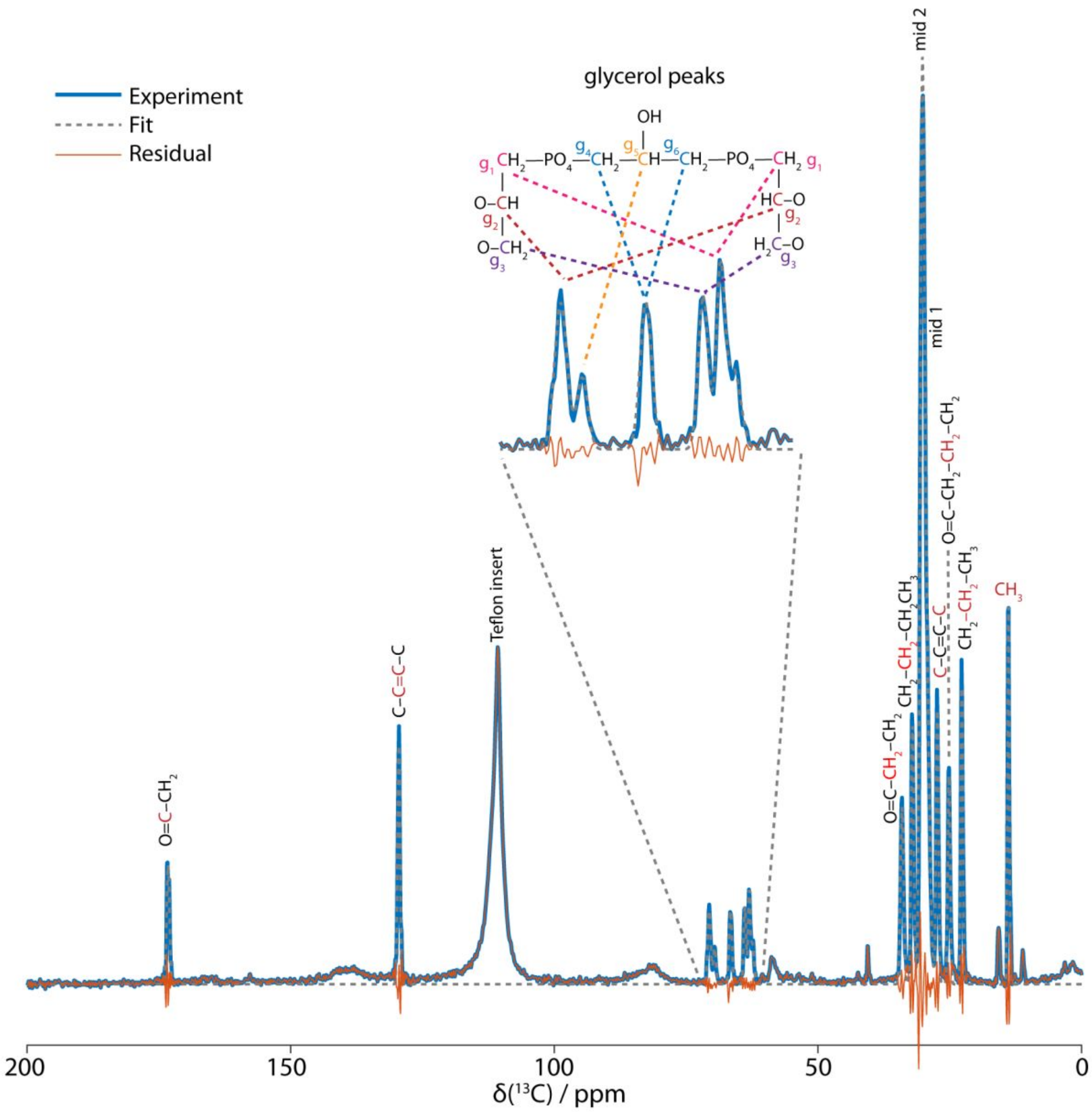

Fig. S6.

^{13}C NMR spectrum assignment and fit for relaxation data analysis. Blue lines give the experimental spectrum, grey dashed lines show the INFOS fit, and the red line gives the residual. Assigned cardiolipin peaks are labeled above the peak, where the red carbon is the assigned peak. Note that some peaks are ambiguous, for example, CH_3, indicates all methyl groups, C–C=C–C are both double bonded carbons, etc.

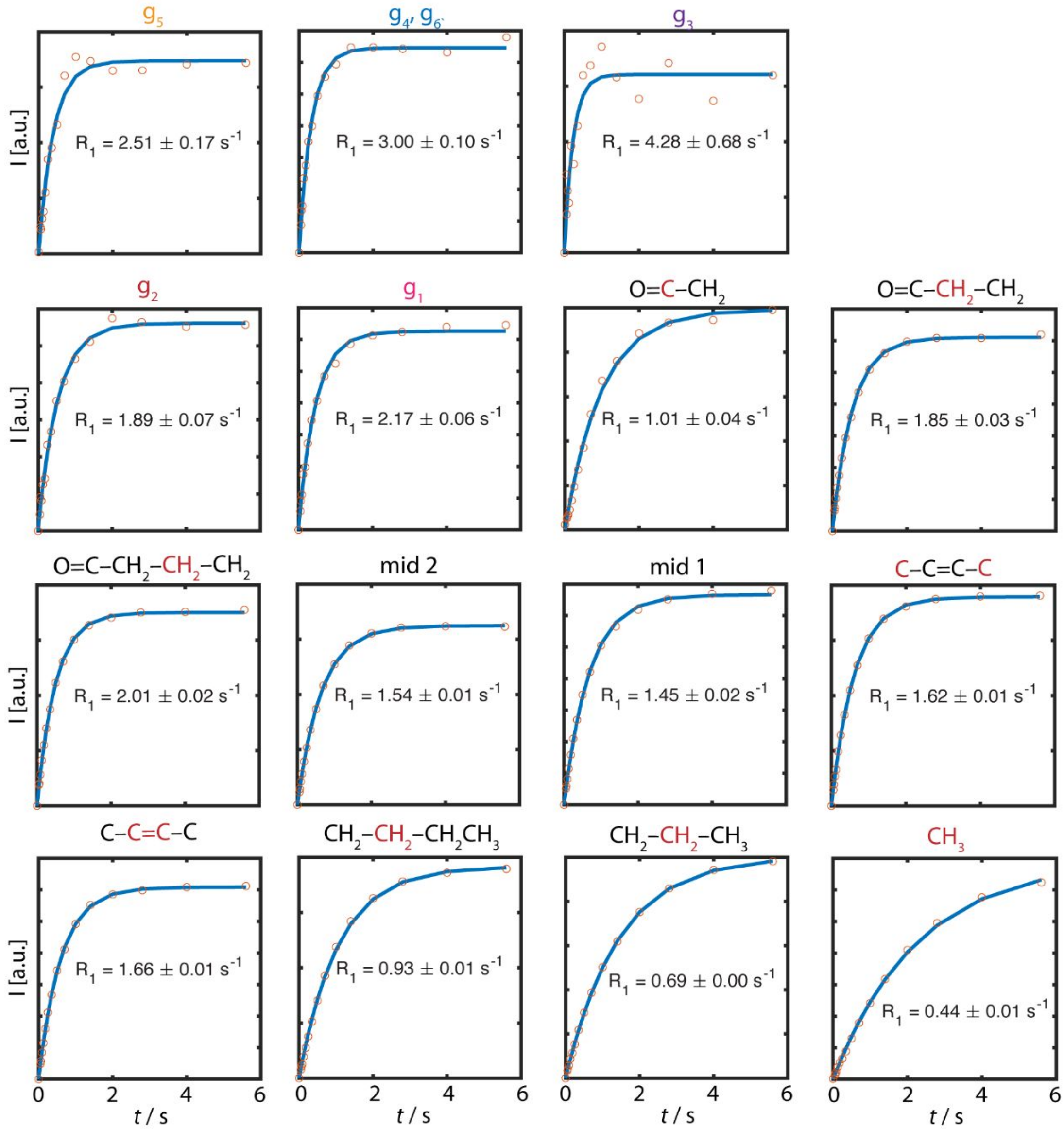

Fig S7. Fits of the individual peaks of CL in POPE/POPG/CL (molar ratio 67.5/22.5/10) in the presence of GlpG and time points to a monoexponential recovery for ^{13}C T_1 relaxation at 16.4 T. Each acquired spectrum in the T_1 series was fitted using INFOS to extract peak amplitudes, and the resulting amplitudes were fitted to a monoexponential recovery curve, $A(1 - \exp(-R_1 t))$, with fit variables A and R_1. Note that these curves are provided to show the fit quality to a monoexponential curve, however, the actual relaxation rate constants were extracted by fitting all spectra simultaneously, with a second pseudo-dimension being fit with the exponential recovery function (FitTrace.m in INFOS). This latter approach uses fewer individual fit parameters, and therefore less noise propagates from the experimental spectra to the final relaxation rate constants.

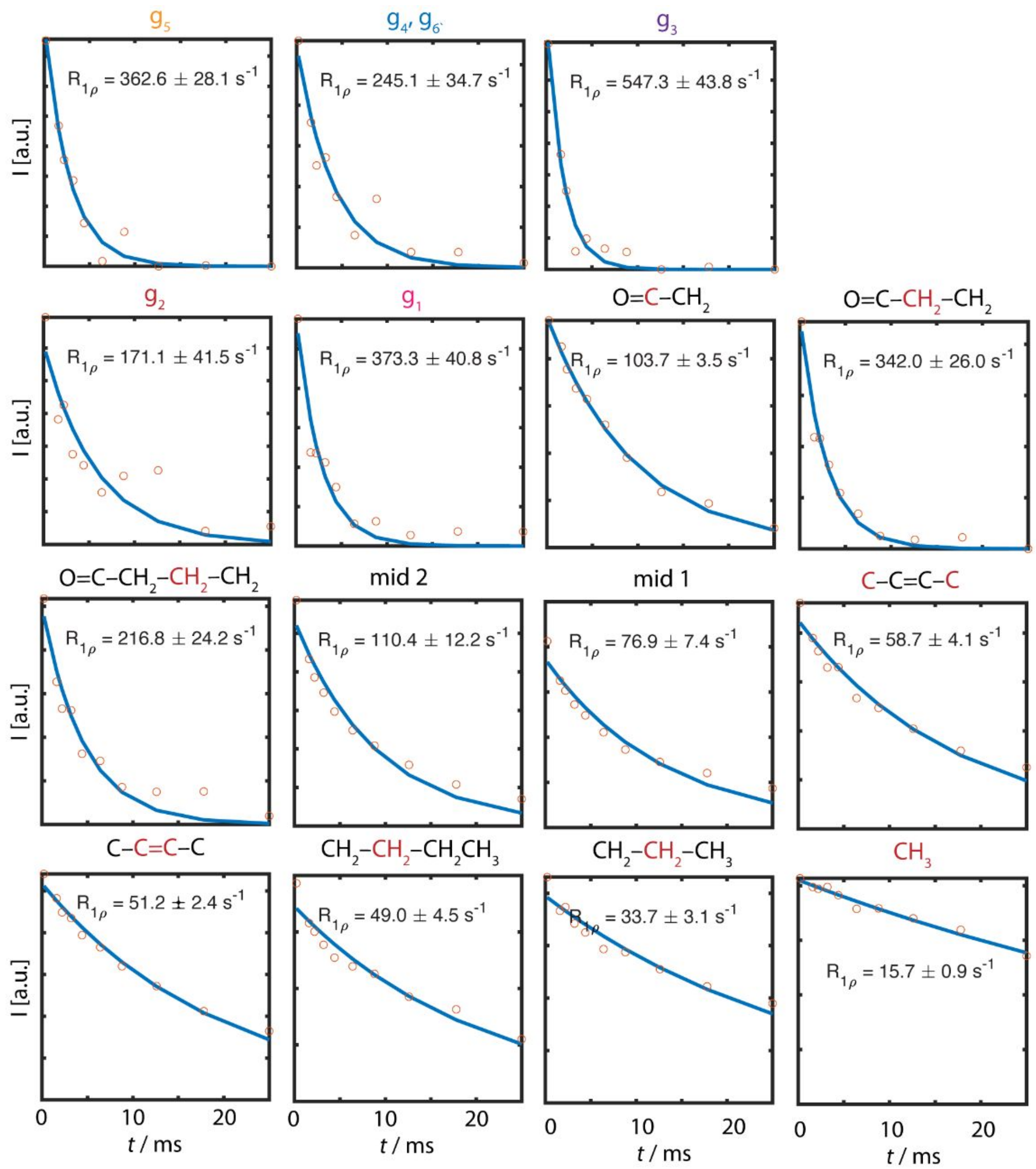

Fig S8. Fits of the individual peaks of CL in POPE/POPG/CL (molar ratio 67.5/22.5/10) in the presence of GlpG and time points to a monoexponential decay for ^{13}C $T_{1\rho}$ relaxation with a spin-lock of 22 kHz. Each acquired spectrum in the $T_{1\rho}$ series was fitted using INFOS to extract peak amplitudes, and the resulting amplitudes were fitted to a monoexponential decay curve, $A \exp\left(-R_{1\rho}t\right)$, with fit variables A and $R_{1\rho}$. These curves are provided to show the fit quality to a monoexponential curve, however, the actual relaxation rate constants were extracted by fitting all spectra simultaneously, with a second pseudo-dimension being fit with the exponential recovery function (FitTrace.m in INFOS). This latter approach uses fewer individual fit parameters, and therefore less noise propagates from the experimental spectra to the final relaxation rate constants. Note that, for $T_{1\rho}$ relaxation when two (or three) ^{1}H are bonded, be expect a biexponential behavior, where if both ^{1}H are spin-up or spin-down, no relaxation will occur initially (since both residual dipole couplings point along the membrane director). However, once one of the ^{1}H flips due to T_1, homonuclear NOE, or coherent spin-diffusion, then $T_{1\rho}$ relaxation of the ^{13}C will resume. Weak monoexponential behavior is observed for a few spins, suggesting relatively fast ^{1}H spin-flips.

Table S1.

^{2}H NMR order parameters of POPE-d_{31} in POPE/POPG mixtures (molar ratio 75/25) with and without 10 mol% CL in the presence and in the absence of GlpG.

Carbon number	w/o CL		w/ CL	
	w/o GlpG	w/ GlpG	w/o GlpG	w/ GlpG
2	0.255	0.217	0.240	0.239
3	0.252	0.203	0.235	0.231
4	0.248	0.193	0.231	0.222
5	0.244	0.183	0.228	0.214
6	0.239	0.171	0.223	0.206
7	0.233	0.161	0.217	0.196
8	0.227	0.152	0.211	0.187
9	0.221	0.142	0.205	0.177
10	0.212	0.131	0.194	0.168
11	0.198	0.119	0.178	0.156
12	0.181	0.106	0.161	0.145
13	0.156	0.091	0.136	0.129
14	0.130	0.076	0.111	0.112
15	0.097	0.053	0.086	0.087
16	0.022	0.0078	0.010	0.025

Table S2.

Experimental parameters for the ^{13}C MAS NMR investigations of U-^{13}C-CL in POPE/POPG membranes in the presence and in the absence of GlpG

Experiment	# Scans	# Replicates	# Time points	# Delay range
Without GlpG				
T_1 (400 MHz)	1024	5	16	100 ms — 11.2 s
T_1 (600 MHz)	768	4	16	60 ms — 8 s
T_1 (700 MHz)	512	6	16	50 ms — 5.6 s
NOE (600 MHz)	2048	3	2 (On+Off)	13 s buildup
NOE (700 MHz)	1024	5	2 (On+off)	10 s buildup
$T_{1\rho}$ (8 kHz)	256	6 (x3 offsets)	14	1.6 ms — 100 ms
$T_{1\rho}$ (12 kHz)	256	6 (x2 offsets)	14	1.6 ms — 100 ms
$T_{1\rho}$ (22 kHz)	256	6 (x2 offsets)	14	1.6 ms — 100 ms
DIPSHIFT (700 MHz)	512	5	17	0 – 200 μs (1 rotor period)
With GlpG				
T_1 (400 MHz)	768	5	16	100 ms — 11.2 s
T_1 (600 MHz)	512	4	16	50 ms — 5.6 s
T_1 (700 MHz)	512	5	16	50 ms — 5.6 s
NOE (600 MHz)	1024	5	2 (On+Off)	13 s buildup
NOE (700 MHz)	1024	6	2 (On+off)	10 s buildup
$T_{1\rho}$ (8 kHz)	256	3 (x3 offsets)	10	1.6 ms — 25 ms
$T_{1\rho}$ (12 kHz)	256	3 (x2 offsets)	10	1.6 ms — 25 ms
$T_{1\rho}$ (22 kHz)	256	3 (x2 offsets)	10	1.6 ms — 25 ms
DIPSHIFT (700 MHz)	512	8	17	0 – 200 μs (1 rotor period)

Table S3.

Relaxation rates and standard deviations for the ^{13}C MAS NMR investigations of U-^{13}C-CL in POPE/POPG membranes in the presence and in the absence of GlpG

Without GlpG									
	NOE (700 MHz)	NOE (600 MHz)	R_1 (700 MHz)	R_1 (600 MHz)	R_1 (400 MHz)	$R_{1\rho}$ (22 kHz)	$R_{1\rho}$ (12.5 kHz)	$R_{1\rho}$ (7 kHz)	S^2
g_5	0.5688	0.3356	2.5516	3.9372	8.6974	28.0140	19.0095	30.0150	0.0000
σ	0.0251	0.0648	0.1127	0.7601	1.2369	7.9828	4.9939	8.6737	0.0017
g_4, g_6	0.3055	0.4515	3.6234	5.0982	6.8537	27.0135	24.0120	36.0180	0.0025
σ	0.0098	0.0320	0.1168	0.3618	0.6754	3.2542	2.5319	6.1903	0.0020
g_3	0.2188	0.3024	3.6234	4.4391	5.6313	47.0235	32.0160	63.0315	0.0259
σ	0.0081	0.0280	0.1350	0.4114	0.5768	7.0581	3.8900	12.6229	0.0010
g_2	0.2225	0.2393	2.1412	2.5282	5.2505	41.0205	31.0155	44.0220	0.0156
σ	0.0058	0.0184	0.0556	0.1948	0.4951	5.1963	2.6964	5.5961	0.0026
g_1	0.1955	0.2423	3.1550	3.2138	7.8557	54.0270	49.0245	68.0340	0.0102
σ	0.0186	0.0152	0.2995	0.2022	0.5721	0.5243	1.3435	8.9623	0.0025
O=C–CH_2	0.2549	0.3040	2.3053	2.6969	3.8076	44.0220	31.0155	61.0305	0.0142
σ	0.0062	0.0119	0.0558	0.1058	0.2324	2.1536	1.2372	5.3277	0.0024
O=C–CH_2–CH_2	0.2891	0.3506	2.1021	2.5049	3.3066	39.0195	15.0075	50.0250	0.0207
σ	0.0087	0.0110	0.0633	0.0786	0.0456	5.3536	1.0038	6.3761	0.0020
mid2	0.2179	0.2331	1.3251	1.4398	1.8838	43.0215	22.0110	50.0250	0.0185
σ	0.0094	0.0022	0.0574	0.0136	0.0290	2.0919	0.6699	2.1086	0.0020
mid1	0.2069	0.2112	1.4667	1.5218	1.6032	11.0055	7.0035	15.0075	0.0098
σ	0.0109	0.0019	0.0771	0.0140	0.0500	0.5812	0.4258	0.8697	0.0008
C–C=C–C	0.2401	0.2736	1.4667	1.5218	1.9038	15.0075	7.0035	18.0090	0.0062
σ	0.0062	0.0103	0.0379	0.0571	0.1366	0.5243	0.4258	1.3090	0.0023
C–C=C–C	0.3505	0.4518	1.4940	1.6085	1.9639	13.0065	6.0030	15.0075	0.0000
σ	0.0108	0.0070	0.0461	0.0251	0.0431	1.3562	0.6928	1.0379	0.0012
CH_2–CH_2–CH_3	0.1505	0.1045	0.8125	0.6333	1.1423	7.0035	7.0035	13.0065	0.0094
σ	0.0042	0.0061	0.0228	0.0372	0.0848	0.8264	0.5846	0.9900	0.0009
CH2–CH3	0.1010	0.1000	0.5775	0.5669	0.7816	7.0035	5.0025	9.0045	0.0003
σ	0.0028	0.0013	0.0157	0.0076	0.0531	0.8467	0.4566	1.1543	0.0008
CH_3	0.0465	0.0420	0.3640	0.3083	0.4008	3.0015	2.0010	5.0025	0.0000
σ	0.0035	0.0033	0.0275	0.0238	0.0221	0.7728	0.4946	0.9604	0.0015

With GlpG									
	NOE (700 MHz)	NOE (600 MHz)	R_1 (700 MHz)	R_1 (600 MHz)	R_1 (400 MHz)	$R_{1\rho}$ (22 kHz)	$R_{1\rho}$ (12.5 kHz)	$R_{1\rho}$ (7 kHz)	S^2
g_5	0.5655	0.4826	2.5050	3.2265	4.5491	661.3307	424.2121	704.3522	0.0000
σ	0.0781	0.0666	0.1099	0.4453	0.3020	116.9364	49.6078	106.7787	0.0017
g_4, g_6	0.2565	0.2642	3.0261	3.2265	4.9699	340.1701	273.1366	175.0875	0.0048
σ	0.0172	0.0177	0.1154	0.2167	0.2435	28.1743	39.2381	22.1393	0.0023
g_3	0.1711	0.2615	4.3687	4.0681	3.8677	389.1946	721.3607	5.0025	0.0164
σ	0.0152	0.0232	0.1996	0.3614	0.2844	131.2424	196.1943	119.4187	0.0014
g_2	0.2419	0.2342	1.9038	1.7836	3.4068	317.1586	204.1021	82.0410	0.0100
σ	0.0443	0.0429	0.0813	0.3269	0.3157	84.4874	58.2913	122.3623	0.0019
g_1	0.2570	0.1786	2.1643	2.8657	6.4930	744.3722	428.2141	340.1701	0.0164
σ	0.0194	0.0135	0.0815	0.2159	0.9961	42.4706	20.4984	73.776	0.0024
O=C–CH_2	0.2317	0.1981	1.8437	2.0641	2.9058	623.3117	425.2126	823.4117	0.0052
σ	0.0054	0.0046	0.0146	0.0482	0.0198	10.9206	2.4396	56.8988	0.0028
O=C–CH_2–CH_2	0.2922	0.2801	2.0040	2.2645	2.9259	335.1676	250.1251	485.2426	0.0076
σ	0.0012	0.0012	0.0040	0.0097	0.0082	21.9101	13.0255	41.4879	0.0022
mid2	0.2408	0.2395	1.5431	1.5030	1.9238	194.0970	108.0540	215.1076	0.0037
σ	0.0022	0.0022	0.0175	0.0139	0.0284	14.8133	2.9260	19.5216	0.0025
mid1	0.1937	0.1899	1.4429	1.5832	1.9238	108.0540	79.0395	174.0870	0.0041
σ	0.0023	0.0023	0.0194	0.0189	0.0536	11.1453	3.0916	23.8060	0.0024
C–C=C–C	0.2425	0.2322	1.6232	1.7034	2.0441	97.0485	62.0310	155.0775	0.0015
σ	0.0028	0.0027	0.0170	0.0199	0.0403	1.1796	1.0412	8.6247	0.0018
C–C=C–C	0.3560	0.3971	1.7435	1.6032	1.9840	85.0425	53.0265	147.0735	0.0000
σ	0.0048	0.0054	0.0173	0.0216	0.0316	2.1878	1.0412	9.8471	0.0016
CH_2–CH_2–CH_3	0.1417	0.1423	0.9419	0.9218	1.1623	62.0310	44.0220	146.0730	0.0011
σ	0.0021	0.0021	0.0051	0.0135	0.0113	3.6466	2.1016	11.3478	0.0021
CH2–CH3	0.1204	0.1167	0.6814	0.7214	0.8016	55.0275	36.0180	90.0450	0.0018
σ	0.0010	0.0009	0.0036	0.0058	0.0078	2.4667	1.4681	5.2383	0.0016
CH_3	0.0500	0.0494	0.4409	0.4208	0.4810	22.0110	14.0070	33.0165	0.0500
σ	0.0003	0.0003	0.0039	0.0022	0.0031	1.1796	1.0778	1.4173	0.0003

Table S4.

Identified cardiolipin (CL) species using electrospray ionization mass spectrometry (ESI MS) of purified CL fraction from *E. coli*.

CL species		Precursor Formula $[M-H]^-$	Exact mass $[M-H]^-$	identified *m/z* value $[M-2H]^{2-}$
CL 64:2		$C_{73}H_{137}O_{17}P_2$	1347.9336	673.5
CL 64:1		$C_{73}H_{139}O_{17}P_2$	1349.9493	674.5
CL 65:2		$C_{74}H_{139}O_{17}P_2$	1361.9493	680.5
CL 65:1		$C_{74}H_{141}O_{17}P_2$	1363.9649	681.5
CL 66:3		$C_{75}H_{139}O_{17}P_2$	1373.9505	686.5
CL 66:2		$C_{75}H_{141}O_{17}P_2$	1375.9650	687.5
CL 66:1		$C_{75}H_{143}O_{17}P_2$	1377.9806	688.5
CL 67:3		$C_{76}H_{141}O_{17}P_2$	1387.9649	693.5
CL 67:2		$C_{76}H_{143}O_{17}P_2$	1389.9806	694.5
CL 67:1		$C_{76}H_{145}O_{17}P_2$	1391.9962	695.5
CL 68:3		$C_{77}H_{143}O_{17}P_2$	1401.9806	700.5
CL 68:2		$C_{77}H_{145}O_{17}P_2$	1403.9963	701.5
CL 68:1		$C_{77}H_{147}O_{17}P_2$	1406.0119	702.5
CL 69:3		$C_{78}H_{145}O_{17}P_2$	1415.9962	707.5
CL 69:2		$C_{78}H_{147}O_{17}P_2$	1418.0119	708.5
CL 69:1		$C_{78}H_{149}O_{17}P_2$	1420.0275	709.5
CL 70:4		$C_{79}H_{145}O_{17}P_2$	1427.9963	713.5
CL 70:3		$C_{79}H_{147}O_{17}P_2$	1430.0119	714.5
CL 70:2		$C_{79}H_{149}O_{17}P_2$	1432.0276	715.5
CL 70:1		$C_{79}H_{151}O_{17}P_2$	1434.0432	716.5
CL 71:4		$C_{80}H_{147}O_{17}P_2$	1442.0131	720.5
CL 71:3		$C_{80}H_{149}O_{17}P_2$	1444.0275	721.5
CL 71:2		$C_{80}H_{151}O_{17}P_2$	1446.0432	722.5
CL 72:4		$C_{81}H_{149}O_{17}P_2$	1456.0276	727.5
CL 72:3		$C_{81}H_{151}O_{17}P_2$	1458.0432	728.5
CL 72:2		$C_{81}H_{153}O_{17}P_2$	1460.0589	729.5
